# *ReScape*: transforming coral-reefscape images for quantitative analysis

**DOI:** 10.1038/s41598-024-59123-2

**Published:** 2024-04-17

**Authors:** Z. Ferris, E. Ribeiro, T. Nagata, R. van Woesik

**Affiliations:** 1 Institute for Global Ecology, Florida Institute of Technology, Melbourne, FL 32901 USA; 2 Department of Computer Science, Florida Institute of Technology, Melbourne, FL 32901 USA; 3 Incorporated Foundation Okinawa Environment Science Center, Urasoe, Okinawa 901-2111 Japan

**Keywords:** Community ecology, Marine biology, Imaging and sensing, Computer science, Software

## Abstract

Ever since the first image of a coral reef was captured in 1885, people worldwide have been accumulating images of coral reefscapes that document the historic conditions of reefs. However, these innumerable reefscape images suffer from perspective distortion, which reduces the apparent size of distant taxa, rendering the images unusable for quantitative analysis of reef conditions. Here we solve this century-long distortion problem by developing a novel computer-vision algorithm, *ReScape*, which removes the perspective distortion from reefscape images by transforming them into top-down views, making them usable for quantitative analysis of reef conditions. In doing so, we demonstrate the first-ever ecological application and extension of inverse-perspective mapping—a foundational technique used in the autonomous-driving industry. The *ReScape* algorithm is composed of seven functions that (1) calibrate the camera lens, (2) remove the inherent lens-induced image distortions, (3) detect the scene’s horizon line, (4) remove the camera-roll angle, (5) detect the transformable reef area, (6) detect the scene’s perspective geometry, and (7) apply brute-force inverse-perspective mapping. The performance of the *ReScape* algorithm was evaluated by transforming the perspective of 125 reefscape images. Eighty-five percent of the images had no processing errors and of those, 95% were successfully transformed into top-down views. *ReScape* was validated by demonstrating that same-length transects, placed increasingly further from the camera, became the same length after transformation. The mission of the *ReScape* algorithm is to (i) unlock historical information about coral-reef conditions from previously unquantified periods and localities, (ii) enable citizen scientists and recreational photographers to contribute reefscape images to the scientific process, and (iii) provide a new survey technique that can rigorously assess relatively large areas of coral reefs, and other marine and even terrestrial ecosystems, worldwide. To facilitate this mission, we compiled the *ReScape* algorithm into a free, user-friendly App that does not require any coding experience. Equipped with the *ReScape* App, scientists can improve the management and prediction of the future of coral reefs by uncovering historical information from reefscape-image archives and by using reefscape images as a new, rapid survey method, opening a new era of coral-reef monitoring.

## Introduction

Coral reefs have supported thousands of marine species for hundreds of millions of years^1,2^ and have facilitated the provisioning of ecosystem goods and services for the past few thousand years^3,4^. In the past four decades, however, coral reefs, and the invaluable goods and services they provide, have been damaged primarily by the rise in climate-change-induced marine heatwaves^5,6^. Understanding and predicting the influence of marine heatwaves and other disturbances on coral reefs relies on the availability of extensive historical data. Yet we lack extensive observational data in remote locations, especially before the first global coral-bleaching event in 1997/98^7,8^. While considerable observational data exist in historical reefscape-image archives, ever since the first image of a coral reef was captured in 1885^9^, these data have remained concealed because the images suffer from perspective distortion which misrepresents the size of organisms. In this study, we developed a computer-vision algorithm, which we call *ReScape*, that removes the perspective distortion from reefscape images—providing the opportunity to revisit innumerable archived reefscape images and increase the historical and geographical data on coral reefs, worldwide.

Photography of the world’s coral reefs began over a century ago with the seminal work by Abercromby^9^, Saville-Kent^10^, and Agassiz^11^. These earliest image plates featured reefscapes of vibrant reef flats exposed at low tide, instilling widespread curiosity to study coral-reef ecosystems. Following the first invention of the self-contained underwater breathing apparatus in 1926^12^, a variety of underwater survey methods were developed to quantify reef patterns and processes^13^. Photoquadrat images of coral reefs using film began with the work of Vevers^14^, Bellamy et al.^15^, and Littler^16^, and the stereoscopic equivalent was introduced by Veron & Done^17^. From the mid-1990s, digital cameras and underwater housings became common^18^. As a result, digital photoquadrats became the industry standard among ecologists because (i) digital memory has a much larger storage capacity and is more efficient and convenient than film, and (ii) they can be readily used for quantitative analysis of the reef condition because top-down-view images do not suffer from perspective distortion^19,20^.

**Figure 1:**
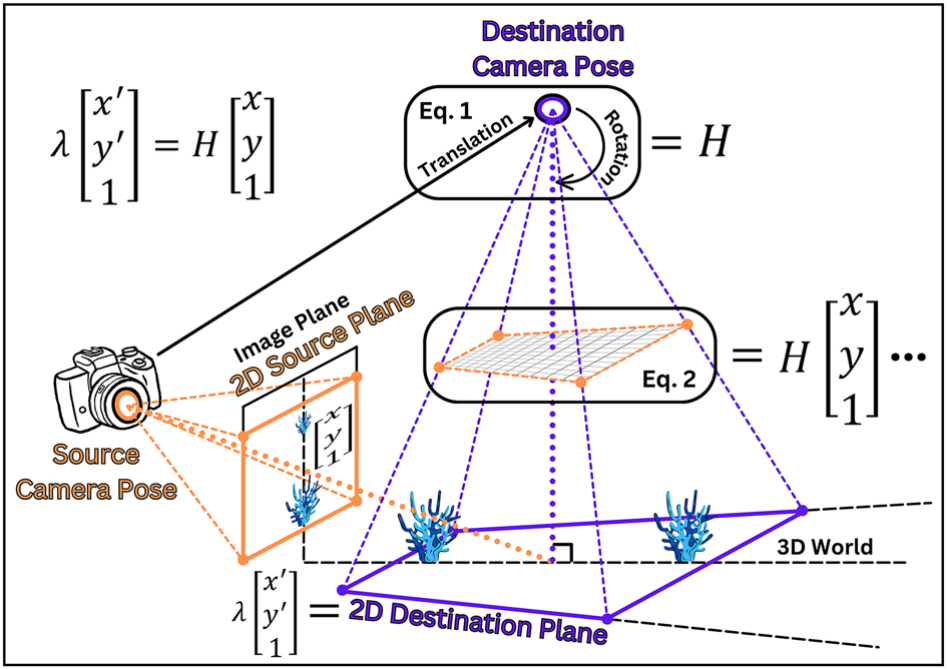
Conceptual schematic of inverse-perspective mapping. The orange and the purple polygons represent the source plane and the destination plane, respectively, which are each defined by four corners with coordinates ($x$, $y$) and (${x}^{\prime}$, ${y}^{\prime}$), respectively. The orange-dashed and the purple-dashed lines radiating from each corresponding camera represent the field of view of the source camera and the destination camera (i.e., pose), respectively. The source camera generates the coral-reefscape image by projecting light received by the three-dimensional (3D) world onto the two-dimensional (2D) image plane. The coral-colony size on the image plane decreases with increasing distance from the source-camera lens. Such perspective distortion arises because the optical axis (orange-dotted line) is not orientated perpendicular to the reef. The source plane is a rectangular selection of the scene to be mapped to the real-world destination plane, producing the transformed top-down view. Therefore, the depicted field-of-view of the source camera is a subset of the total field-of-view. The destination plane is a trapezoid because the source camera’s field-of-view expands with increasing distance from its lens. After the source-camera pose is translated and rotated above the scene in the 3D world (Eq. (1)), the destination plane’s image matrix can be interpolated from the source plane’s image matrix (Eq. (2)), where the three dots indicate computation time, to generate the top-down view of the source plane, where λ and 1 are scaling parameters. This mapping process of transforming the image from the source plane to the destination plane is called transformation homography (*H*) and is parameterized by defining the coordinates of the four source corners and each of their corresponding destination corners. Note that the optical axis of the destination-camera pose (purple-dotted line) is orientated perpendicular to the reef, thereby removing the perspective distortion. The camera-roll angle is zero for visual simplicity.

While digital photoquadrats are regularly used by ecologists to assess the condition of coral reefs, by quantifying the abundance, size, and percent coverage of many reef taxa, capturing reefscape images is preferred by recreational photographers because they are more aesthetically pleasing—inadvertently capturing relatively large areas of reef. Although reefscape imaging of exposed reef flats began over a century ago^9^, it was not until the mid-1990s when recreational divers began exploring the underwater environment with digital cameras with high-storage capacity which proliferated the rate of reefscape-image collection around the world. Underwater reefscape images are captured at an oblique angle to the reef, producing an image with a large expanse of a reef in the foreground and the water column in the background. However, despite their prevalence, reefscape images have not been used to accurately quantify the conditions of reefs because of perspective distortion, which causes the apparent size of reef taxa to decrease with increasing distance from the camera lens (Fig. 1).

In addition to perspective distortion, reefscape images have historically not been conducive to quantitative analysis^21–23^ because the images may be biased toward more aesthetically pleasing areas of reef and not systematically captured through space or time^23^. For these reasons, Wachenfeld^23^ reasoned that “without complicated geometric analysis of the photograph, the best that can be achieved is a qualitative, subjective impression of the substratum shown in the photograph” and in further writing, asserted that “comparisons between historical photographs and modern reef-flats can never provide definitive, stand-alone proof one way or the other in the debate over whether or not the [Great Barrier Reef] is undergoing a steady decline.” In other words, Wachenfeld^23^ perceived that ecological data cannot be extracted from reefscape images to quantify past reef dynamics with sufficient rigor. Yet, such a geometric analysis would be invaluable to ecologists because it would unlock the vast historical information of reef conditions that have been concealed in reefscape-image archives for over the past century.

After receiving a set of thousands of archived coral-reefscape images taken on the reefs of Okinawa, Japan, from 2004 to 2021, we were motivated to develop a geometric analysis that accurately transforms the perspective of reefscape images into top-down views to remove the perspective distortion and display all reef taxa at their actual size. We were inspired by the research-and-development sector of the autonomous-driving industry, because distortionless, real-time mapping of the vehicle’s surrounding environment is required to maximize passenger safety^24,25^. The mapping of the vehicle’s surrounding environment can be powered by the perspective transformation of a forward-facing road image into a top-down view. The transformation technique is called inverse-perspective mapping^26^ and requires a known camera position and orientation (i.e., pose)^27^. While the camera pose is known for autonomous vehicles, the camera pose is unknown for reefscape imagery. Here, we extend inverse-perspective mapping by developing the *ReScape* algorithm to accommodate unknown camera poses.

The objectives of this study were to (i) overcome the problem of unknown camera pose for inverse-perspective mapping by designing and developing the *ReScape* algorithm, (ii) transform the perspective of reefscape images to make them available for quantitative analysis of reef conditions, and (iii) test the *ReScape* algorithm on numerous historical reefscape images collected in Okinawa, Japan. The mission of the *ReScape* algorithm is to unlock the immense historical information about coral reefs that have been concealed in reefscape-image archives for over the past century. The *ReScape* algorithm provides a method to extract historical data from previously unquantified localities and enables citizen scientists and recreational photographers to contribute reefscape images to the scientific process. The *ReScape* algorithm also provides a new survey technique that can rigorously assess relatively large areas of coral reefs, and other marine and even terrestrial ecosystems, worldwide.

## Methods

### Inverse-perspective mapping for ecology

Here, inverse-perspective mapping is the task of transforming the perspective of a reefscape image to recover its representative top-down view. The original reefscape image contains the two-dimensional (2D) source plane, which is mapped to the 2D destination plane in the three-dimensional (3D) world to produce its transformed top-down view (Fig. 1).

The source plane and the destination plane are each defined by four coordinates that form a polygon. The source plane is a rectangular selection of the desired region to be transformed in the reefscape image. The region of the reefscape image that is not included in the selection of the source plane is ignored during the transformation. The destination plane is a trapezoid because the camera’s field of view expands with increasing distance from the camera lens and therefore must account for the difference of the real-world distance of the top and bottom borders of the source plane.

Conceptually, the mapping process between the coordinates defining the source plane and the destination plane involves finding the required amount of camera translation (i.e., relocation) and rotations (i.e., manipulated yaw, pitch, and roll angles) to position it above the scene such that it is oriented perpendicular to the destination plane and produces the top-down view of the source plane. This mapping process is called transformation homography^28^ and is governed by the perspective transformation matrix, $H$, as

1 $$H=\left[\begin{array}{ccc}{h}_{1}& {h}_{2}& {h}_{3}\\ {h}_{4}& {h}_{5}& {h}_{6}\\ {h}_{7}& {h}_{8}& 1\end{array}\right],$$

where ${h}_{1}$ to ${h}_{8}$ are the parameters that control the camera-pose translation and rotations, and 1 is a scaling parameter. If the camera pose that captured the reefscape image is known, the source- and the destination-corner coordinates can be directly derived, which are then used to compute $H$ directly^27,29^.

**Figure 2:**
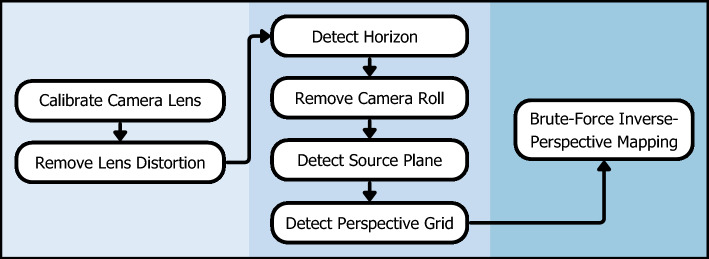
The seven functions of the *ReScape* algorithm for transforming reefscape images into a top-down view make them usable for quantitative analysis of reef conditions. Each function represents a step of the image transformation process for the *ReScape* algorithm. Calibrating the camera lens is only performed once per camera-lens model. The functions on the left preprocess the images, the functions in the middle detect and characterize each scene’s perspective geometry, and the function on the right searches for the transformation homography.

However, because reefscape imagery was not originally intended for quantitative analysis of reef conditions, the camera pose was never recorded, so the source and destination coordinates and $H$ cannot be directly computed. Therefore, defining the coordinates of the source plane and the destination plane to compute $H$ with an unknown camera pose required the design and development of the *ReScape* algorithm, which has seven functions (Fig. 2). After the source and destination coordinates are defined and $H$ is computed, the image matrix defining the source plane can then be multiplied by $H$ to interpolate the image matrix for the destination plane, as

2 $$\lambda \left[\begin{array}{c}{x}^{\prime}\\ {y}^{\prime}\\ 1\end{array}\right]=H\left[\begin{array}{c}x\\ y\\ 1\end{array}\right],$$

where $x$ and $y$ are the pixel coordinates of the source corners, ${x}^{\prime}$ and ${y}^{\prime}$ are their corresponding destination-pixel coordinates, and $\lambda$ is a scaling factor.

### Calibrate camera lens and remove lens distortion

The convex shape of camera lenses inherently introduced considerable radial distortions (i.e., stretching)^30,31^ toward the borders of the reefscape images (Supplementary Fig. S1). Additionally, because of the imperfect manufacturing of cameras, the camera lens and sensor were not perfectly parallel, which introduced subtle tangential distortions (i.e., decentering)^31^ in the reefscape images (Supplementary Fig. S1). Therefore, these lens distortions in the reefscape images were removed to (i) generate a better representation of reality, (ii) improve the determination of the scene’s perspective geometry, and (iii) promote accurate calculations of the surface areas and sizes of coral colonies after the perspective transformation. This involved structure-from-motion imaging of a static calibration pattern in a shallow reef environment to determine our camera’s (Olympus Tough TG-6) intrinsic calibration parameters (i.e., camera matrix) and distortion coefficients using well-established functions^32^. Because the focal-length parameters in the camera matrix are sensitive to magnification, which was caused by the incoming light transitioning from the seawater to the small volume of air between the camera’s lens and waterproof housing (i.e., changing the refractive index)^33^, the camera-calibration-imaging process was performed underwater, while using the default zoom of 1.0X (i.e., the same zoom that is conventionally used for underwater photography). Then, the camera matrix for each reefscape image was refined to remove the lens distortions from each image (Supplementary Fig. S1). Because the removal of the lens distortions introduced an absence of field-of-view along the image borders, the maximum inscribed, axis-aligned rectangle was then extracted from the reefscape images without camera-lens distortions for further processing, which slightly reduced the dimensions of the image (Supplementary Fig. S1). Over two-hundred camera-lens-calibration images were used to obtain an assessment of the calibration pattern (i) from different angles and (ii) composed in different areas throughout the image frame (i.e., not just in the middle of the image frame).

Radial- and tangential-lens distortions vary at the micron scale for manual adjustments in the focal length of a given camera lens^34^. Therefore, changes in the *effective* focal length from the subtly varying refractive index of the seawater (i.e., spatio-temporally varying temperature, salinity, suspended particles) and the small volume of air in the camera housing (i.e., temporally-varying temperature) would produce a trivial error. Therefore, the camera-calibration-imaging process was performed once for the Olympus Tough TG-6 camera several years after the historical reefscape images were captured. While not necessary for successful calibration, the technical reader can account for these micron-scale variations by calibrating their camera before every survey.

### Detect horizon

The horizon line was detected in the reefscape images because it was the basis of all subsequent detections and characterizations of their perspective geometries (Fig. 2). It was apparent that detecting edges in the reefscape images could demarcate their horizon lines because the water columns produced no edges, whereas the texture of the reefs produced many edges (Supplementary Fig. S3). Edge-detection algorithms are rooted in the expectation that the pixel-intensity value will rapidly change across object borders because of the color contrast between objects^35^. Therefore, the reefscape images were converted to greyscale to isolate their intensity channels (Supplementary Fig. S3).

Canny-edge detection^36^ is an algorithm that, in brief, computes the intensity gradient at each pixel and then classifies the pixel as an edge if its intensity gradient is above a user-defined threshold. To promote accurate computations of the intensity gradient in the greyscale-reefscape images, a Gaussian blur was applied to remove high-frequency noise^37^ caused by unpredictable light scattering and absorption by water molecules and particulate matter^38^ (Supplementary Fig. S3). Because the gradient threshold for Canny-edge detection is based on all the intensity values in the Gaussian-blurred-reefscape images, faint edges along the horizon lines were not detected owing to the lack of light in the background of the images, which caused their intensity gradients to be relatively low. Therefore, before Canny-edge detection, the Gaussian-blurred-reefscape images were binarized into foreground and background pixels using adaptive thresholding^39^ (Supplementary Fig. S3).

For each Gaussian-blurred-reefscape image, adaptive thresholding enabled the classification of faint intensity gradients as foreground pixels because the intensity thresholds were computed for each pixel based on the intensity values within a local neighborhood instead of all the intensity values in the image. That is, adaptive thresholding classified background pixels, near the horizon lines, as foreground pixels because they were not weighed against the comparatively higher-intensity gradients in the foreground of the Gaussian-blurred-reefscape images. For each reefscape image, the desired ‘edge map’ with detected edges, right up against the horizon line, was produced by applying Canny-edge detection on the adaptively-thresholded image (Supplementary Fig. S3).

For each edge map, the density of edge pixels at each pixel was then computed within a local neighborhood and normalized within the range of 0 to 1 to produce the ‘edge-density map’ (Supplementary Fig. S3). For each edge-density map, the reef produced a comparatively higher density of edge pixels than the water column, and it was expected that the horizon line would follow this demarcation of the density of edge pixels (Supplementary Fig. S3). However, if the water surface was visible in the original reefscape image, the presence of aquatic life superimposing the water column caused the horizon demarcation to ‘bleed’ into the water surface because these features also produced high-edge densities (Supplementary Fig. S3). Therefore, to remove these features and make the aquatic life behave as noise, one-hundred pixels, with zero-edge density that were within the region of the water column, were randomly sampled from each edge-density map (Supplementary Fig. S3). However, corals cast shadows that also have zero-edge density, which were subject to inclusion during the random sampling (Supplementary Fig. S3). Therefore, the Theil-Sen estimator, a robust linear-regression algorithm^40,41^, was used to regress the randomly sampled pixels and obtain the slope and y-intercept of the ‘water-column line’ in each edge-density map (Supplementary Fig. S3).

The Theil-Sen estimator iteratively computes the regression parameters for every possible line created by a unique pair of data points and returns the median values, thereby being insensitive to outliers. Accordingly, the Theil-Sen estimator does not require any data-set-specific hyperparameter tuning, which is desired for consistently resolving the variable position of the water-column line in the reefscape images. Every pixel above the water-column line was then reassigned to zero to make the water-surface region in each edge-density map ‘invisible’ to the computer (Supplementary Fig. S3). If the water surface was not visible in the original reefscape image, this step simply removed the excess water column.

The grey-level histogram of edge-pixel densities for each reefscape image was primarily bimodal, with a pronounced signal near 0 and a broader signal toward 1, representing the remaining water column and reef, respectively (Supplementary Fig. S3). The optimal Otsu threshold that best separated these two signals^42^ was then found by minimizing the intra-class variance of each edge-density-map’s intensity channel (Supplementary Fig. S3). Otsu thresholding allowed us to binarize the intensity channel of the edge-density map into reef and water column regions (Supplementary Fig. S3). Contours were then detected^43^ in each Otsu-thresholded image and the longest contour was selected, which strictly followed the horizon line and wrapped around the image border (Supplementary Fig. S3). Points from the longest-selected contour that laid along the border of the Otsu-thresholded images were removed (Supplementary Fig. S3). A z-score outlier filter was applied on the y-coordinate of the remaining points for each reefscape image (Supplementary Fig. S3). Any remaining outliers were a result of the absence of detected edges within shadows near the border of the Otsu-thresholded images (Supplementary Fig. S3). The Theil-Sen estimator was used again to linearly regress the remaining contour points to obtain the slope and y-intercept of the horizon line for each reefscape image for the same reasons as the water-column-line regression (Supplementary Fig. S3).

### Remove camera roll

The camera-roll angle was removed in each reefscape image to eliminate perspective bias along the horizontal axis because this greatly simplified the search space for the source and destination corners. Specifically, with a non-zero camera-roll angle, the bottom-source corners (and the top-source corners) do not have an equidistant mapping to their respective destination corners, requiring a two-dimensional search—the two dimensions being (i) the distance of each destination corner from the camera and (ii) the distance between each destination corner. Removing the camera-roll angle made the two closest destination corners to the camera equidistant from the camera (as well as the two furthest destination corners), as depicted in Fig. 1, reducing the search to a one-dimensional problem. Next, only the distance between destination corners had to be found for each reefscape image (see *Brute-Force Inverse-Perspective Mapping*).

The camera-roll angle was removed by rotating each reefscape image about the image center by the opposite of the horizon-line slope (Supplementary Fig. S4). Upon rotation, thin black margins were padded along each image border where there was no field-of-view (Supplementary Fig. S4). Therefore, the largest inscribed, axis-aligned rectangle was extracted from each rotated reefscape image for all subsequent image manipulations, which also slightly reduced the image dimensions (Supplementary Fig. S4). For each reefscape image, the y-intercept of the horizon line was updated according to rotational geometry and the amount of vertical cropping.

### Detect source plane

The transformable reef plane was detected in each reefscape image to define the source plane for the perspective transformation. The bottom-left and bottom-right corners of the image itself were selected for the bottom-source corners. However, for the top-source corners, the top-left and top-right corners of the image itself needed to be moved by an equivalent vertical amount to a position below the horizon line. Otherwise, if the top-source corners were superimposed on the water surface or the water column, then an undesired plane would have been created running through the height of the water column or an infinite plane, respectively. If these cases were to be perspective transformed, the former would result in an ‘under-corrected’ camera-pitch angle, while the latter would never converge to a top-down camera-pitch angle.

To locate the y-coordinate of the top-source corners for each reefscape image, the row below the horizon line was detected resulting in the largest source plane while minimizing the introduction of new distortions after the perspective transformation^44^ (Supplementary Fig. S5). That is, where excessive perspective distortion occurred along the y-axis toward the background of each reefscape image, this portion of the reef could be ignored because it would respond poorly to the inverse-perspective mapping^44^. Additionally, because the reef covered more area from the camera’s expanding field of view increasingly toward the top of each reefscape image, this portion of the reef was cropped out because it considerably deviated from the flat-source-plane assumption of inverse-perspective mapping. For each reefscape image, this row index was searched for by computing the anisotropy of the standard edge map (i.e., with no adaptive thresholding) within a moving window (Supplementary Fig. S5). Anisotropy in the context of image processing is a measure of the directional dependence of texture features along their major and minor axes^45^ and was computed as

3 $$A={\left[{\sigma }^{2}\left(arctan\left\{\nabla {E}_{n}\right\}\right)\right]}^{-1},$$

where $A$ is the anisotropy and $\left\{\nabla {E}_{n}\right\}$ is a list of all the edge gradients. Therefore, anisotropy here is the reciprocal of the variance of edge orientations. The anisotropy equation was constructed in this way because it was expected that edges toward the horizon would have near-zero slopes because of perspective distortion, translating to high anisotropy values, whereas edges closer to the camera would have slopes drawn randomly from the domain −90° to 90°, translating to low anisotropy values (Supplementary Fig. S5).

Textures with low anisotropy indicated that the degree of perspective distortion was relatively low and should respond well to the perspective transformation. The moving window that calculated anisotropy covered the entire width of each edge map and had a height equal to one-fifth of the distance between the horizon line and the bottom of each edge map (Supplementary Fig. S5). This window was moved downward row-by-row starting from the horizon line to halfway toward the bottom of each edge map (Supplementary Fig. S5). Processing down to the bottom of each edge map was unnecessary because the excessive perspective distortion would occur closer to the horizon line, allowing for a more computationally efficient search (Supplementary Fig. S5).

Anisotropy and the row index were both normalized within the range of 0 to 1. Anisotropy was plotted against the row index starting from the horizon and down halfway toward the bottom of each edge map (Supplementary Fig. S5). The row index was selected where the derivative of the anisotropy curve in normalized space was equal to −1, indicating that the camera was beginning to favor the detection of less anisotropic-texture features (Supplementary Fig. S5). For each edge map, this ‘anisotropy index’ identified the row below the horizon at which rapid information loss began, and which was too distant from the camera and would not respond well to the perspective transformation (Supplementary Fig. S5). Therefore, for each reefscape image, the anisotropy index was selected as the y-coordinate for the top-source corners and this selection therefore finished defining the source plane.

### Detect perspective grid

The perspective geometry of each source plane was characterized by detecting an ‘axis-aligned perspective grid.’ The axis-aligned perspective grid was defined as the highest-ranking vanishing line with a line drawn parallel to the horizon (i.e., horizontal to the x-axis because the camera-roll angle was removed) through its y-coordinate midpoint and this line allowed us to find the optimal destination plane (as detailed in *Brute-Force Inverse-Perspective Mapping*).

The vanishing-line-detection process for each reefscape image began by constructing lines from the standard edge map using the probabilistic Hough-line transform^46^ (Supplementary Fig. S6). All the edges above the horizon line of each edge map that were produced by aquatic life or surface reflections were removed before the line-detection process (Supplementary Fig. S6). Because our users will be working in an extensive variety of habitats, potentially also in habitats with low coral cover, the constraints of the line detection were relaxed to allow the detection of many small lines (Supplementary Fig. S6). Logical-angle filtering was then applied to all the detected lines in each edge map according to the y-intercept of the horizon line (Supplementary Fig. S6). That is, edge maps with a horizon line around the middle third of the image produced vanishing lines that should have converged toward their vanishing point with a shallower angle. By contrast, edge maps with a horizon line toward the top third of the image produced vanishing lines that should have converged toward their vanishing point with a steeper angle.

The angle-filtered lines that were detected in each reefscape image were then grouped based on their x-coordinate midpoint into left lines and right lines (relative to the midpoint of the image width) and further filtered based on their sign to isolate ‘candidate-vanishing lines’ (i.e., vanishing lines on the right side of the image should have numerically positive slopes and vice versa; Supplementary Fig. S6). The group with more candidate-vanishing lines was randomly downsampled to match the number of lines in the group with fewer lines to avoid side-based bias in the downstream ‘candidate-vanishing point’ calculations.

For each reefscape image, the intersections were computed for each unique combination of a left and a right line (Supplementary Fig. S6). These intersections were then filtered for each reefscape image to only include those that lay along the horizon line (Supplementary Fig. S6). These intersections were then further filtered for each reefscape image to only include those within 25% of the image width centered on the image width, isolating ‘candidate-vanishing points’ (Supplementary Fig. S6). These candidate-vanishing points were averaged for each reefscape image to produce the overall-vanishing point (Supplementary Fig. S6). For each reefscape image, the highest-ranking vanishing line was selected from all the left and right lines by computing their perpendicular distances from the overall-vanishing point and selecting the minimum one (Supplementary Fig. S6). The y-coordinate midpoint of each reefscape image’s selected-vanishing line was constrained below the anisotropy index to ensure that the selected-vanishing line was on the source plane. If the reefscape image’s selected-vanishing line was shorter than five-hundred pixels, its length was extended to five-hundred pixels to ensure it remained ‘visible’ to the computer throughout the homography search (see *Brute-Force Inverse-Perspective Mapping*). The axis-aligned perspective grid was then formed for each reefscape image by drawing a horizontal line across the entire width of the image through the y-coordinate midpoint of the selected-vanishing line (Supplementary Fig. S6).

### Brute-force inverse-perspective mapping

At this stage of the *ReScape* algorithm, all four corners of the source plane have been defined, the axis-aligned perspective grid has been drawn on the source plane, and the source plane has been extracted from each reefscape image in preparation for the perspective transformation. The definition of the destination plane for each reefscape image began by setting the two furthest destination corners from the camera equal to the top-source corners. To find the transformation homography for each reefscape image, the distance between the two closest destination corners to the camera was reduced to reflect the increasing field-of-view toward the top-of-the-source plane. The optimal distance reduction was found for each reefscape image by searching for a compression factor that was multiplied by the width of the source plane. That is, for each reefscape image, to define the closest destination corners, the amount of horizontal shift was found by re-positioning the bottom-left and bottom-right source corners toward the center of the source plane (see Supplementary Animation). Thus, for each reefscape image, the ratio of the real-world distance of the top border to the bottom border of the source plane can be searched for mathematically, as follows

$$d={x}_{cf}w$$

$${C}_{L}=\left(\frac{w}{2}-\frac{d}{2},h\right)$$

4 $${C}_{R}=\left(\frac{w}{2}+\frac{d}{2},h\right),$$

where $d$ is the horizontal-pixel distance between the two closest destination corners to the camera, ${x}_{cf}$ is the compression factor, $w$ is the width of the source plane, ${C}_{L}$ and ${C}_{R}$ are the coordinates of the closest left-side and right-side destination corners, respectively, and $h$ is the pixel height of the source plane. For each reefscape image, a brute-force approach searched for ${x}_{cf}$ by iteratively transforming the source plane while decreasing ${x}_{cf}$ by a small amount throughout its entire parameter space (0 < ${x}_{cf}$ < 1) (Supplementary Fig. S7). For each search iteration, the following procedure was applied to each reefscape image: (i) the perspective grid was segmented in hue, saturation, and value color space, (ii) morphological closing was applied to the segmented mask to reduce any noise^47^, (iii) the mask’s edges and lines were detected, and (iv) the average angle between the detected lines of the mask was computed (Supplementary Fig. S7). For each reefscape image, the brute-force search for ${x}_{cf}$ (i.e., searching for the transformation homography) stopped and saved the value of ${x}_{cf}$ when a perspective-grid angle equal to 90° was detected, indicating that the vanishing lines had become parallel to each other and therefore all perspective-induced distortions had been removed (Supplementary Fig. S7)—thus successfully creating the optimal top-down view of reefscape images.

The detected parallelization of vanishing lines, as indicated by the 90° perspective grid, is the ‘heart’ of the *ReScape* algorithm and is analogous to lane markings becoming parallel to each other in the top-down view of road scenes for autonomous vehicles. For example, suppose you are looking straight down onto a road from a low-flying airplane. While the drivers on the road would perceive the lane markings as converging toward the vanishing point on the horizon (i.e., the driver’s perspective being ‘*the camera-pose field-of-view that captured the reefscape image*’), you in the airplane would instead be perceiving the lane markings as parallel to each other (i.e., your perspective being ‘*the translated and rotated camera-pose field-of-view that produced the top-down view*’). This realization is what made the *ReScape* algorithm a reality.

### Testing

One hundred and twenty-five historical reefscape images collected around the coastline of Okinawa, Japan, at depths between 0 and 6 m, were processed to determine the process rate and success rate of the *ReScape* algorithm. Only images that were readily apparent to be compatible with the assumptions of brute-force inverse-perspective mapping were processed (i.e., had a horizon line, low to moderate reef rugosity, and good visibility). The process rate was defined as the proportion of reefscape images that were fully processed by the *ReScape* algorithm (i.e., those which raised no errors), whereas the success rate was defined as the proportion of fully processed reefscape images that were accurately transformed by the *ReScape* algorithm into a top-down view.

### Validation

Two landscape images and their ground-truthed top-down views were captured using a DJI Mini 2 drone (Supplementary Fig. S8). For the first landscape image that was captured at an altitude of 16 m, two reference targets were placed towards the bottom of the image frame such that a line segment of 27.07 m was created that ran parallel to the x-axis of the image (Supplementary Fig. S8). This process was repeated for four additional pairs of targets placed at an increasing distance away from the bottom of the image (Supplementary Fig. S8). The pixel distance of each line segment was measured in the landscape image, its transformed top-down view, and its ground-truthed top-down view and was normalized to the width of its corresponding image in pixels. This process provided data to quantify perspective distortion in the x-direction in each image. For the second landscape image, which was also captured at an altitude of 16 m, ten reference targets were equally spaced apart by 3 m along a line, forming nine segments of lines, which ran parallel to the y-axis of the image (Supplementary Fig. S8). The pixel distance of each line segment was measured in the landscape image, its transformed top-down view, and its ground-truthed top-down view and was then normalized to the height of its corresponding image in pixels, allowing for the quantification of perspective distortion in the y-direction in each image. Camera-lens calibration images for the drone were captured in the laboratory.

### The ReScape app

The *ReScape* algorithm was written in Python 3.11.3 using the PyCharm Community Edition interactive development environment. The development of *ReScape* heavily relied on functions provided by the OpenCV^48^, NumPy^49^, Matplotlib^50^, Scikit-learn^51^, and SciPy^52^ external libraries. The *ReScape* algorithm was refactored and compiled into a free, user-friendly App to make it readily accessible to anyone with an internet connection and a Windows computer or a Mac computer running the Windows operating system. The *ReScape* App is available for download at the *ReScape* GitHub repository (see Data Availability). The *ReScape* App is initialized by the user (1) selecting a folder containing reefscape images, (2) selecting a folder for saving the transformed images, (3) submitting the percentage of central-processing-unit resources to allocate, (4) submitting the name of their camera model, (5) selecting a folder for saving camera-lens-calibration parameters, and (6) selecting a folder containing the camera-lens-calibration images. After these six initialization steps, the *ReScape* App dynamically removes the perspective distortion from reefscape images captured by any standard camera. Steps 4–6 are not needed if the user selects camera-lens-calibration parameters that were automatically saved by a previous run because any given camera-lens model only needs to be calibrated once. No coding experience or installation of any other software is required to use the *ReScape* App. Users who wish to modify the *ReScape* source code can access it at the *ReScape* GitHub repository (see Data Availability).

## Results

**Figure 3:**
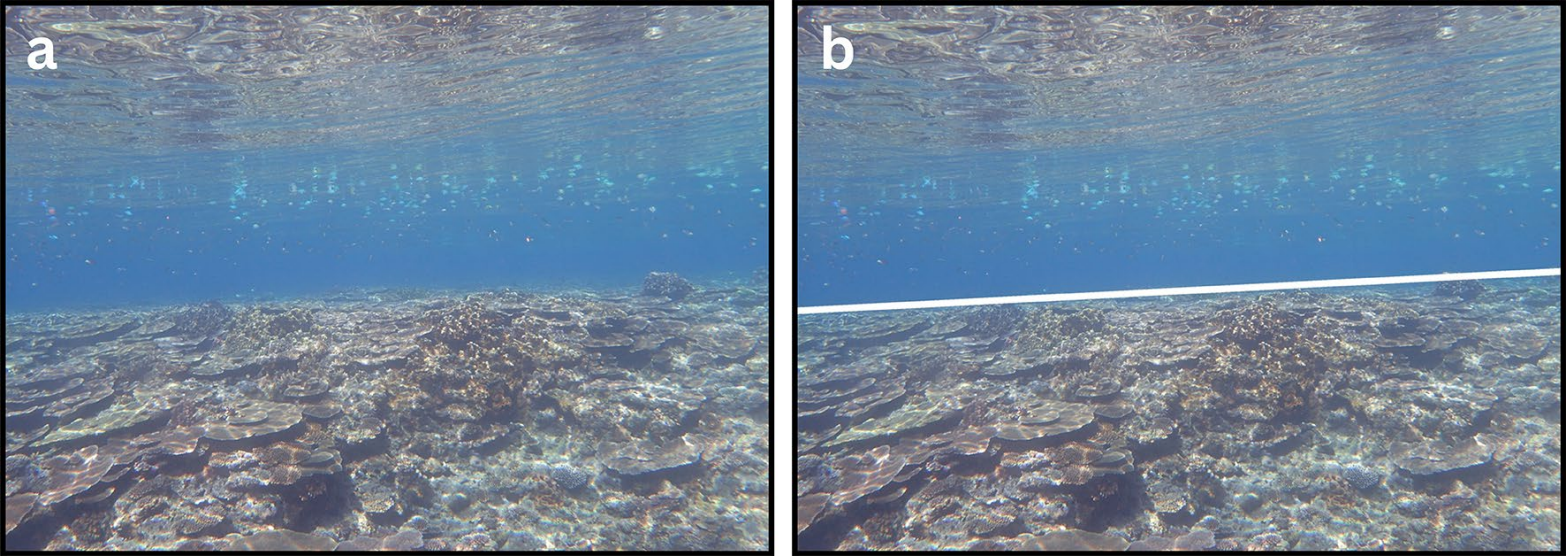
Two images demonstrate the effect of the Detect-Horizon function, as shown in Fig. 2. (**a**) The image without camera-lens distortions before horizon-line detection and (**b**) after horizon-line detection. Note that the detected horizon line (white line) in (**b**) is where the reef plane vanishes toward the background of the image in (**a**). The intermediate-image manipulations are shown in Supplementary Fig. S3.

**Figure 4:**
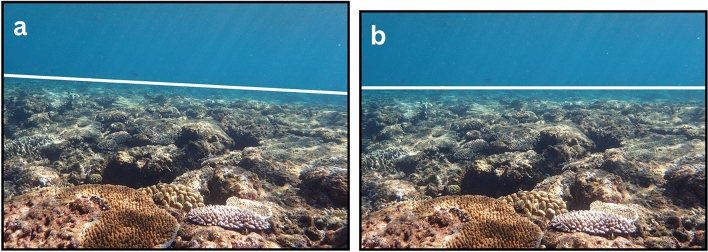
Two images demonstrate the effect of the Remove-Camera-Roll function, as shown in Fig. 2. (**a**) The image without camera-lens distortions before removing the camera-roll angle and (b) after removing the camera-roll angle. Note that the horizon line (white line) in (**a**) became parallel to the x-axis in (**b**). Also, note how the image dimensions in (**b**) are slightly reduced relative to (**a**), owing to the narrower axis-aligned field of view. The intermediate-image manipulations are shown in Supplementary Fig. S4.

**Figure 5:**
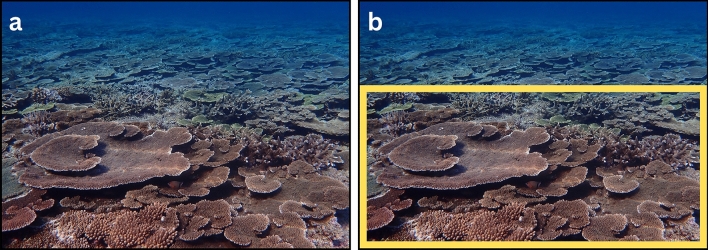
Two images demonstrate the effect of the Detect-Source-Plane function, as shown in Fig. 2. (**a**) The rotated image before detecting the source plane and (**b**) after detecting the source plane. Note that the top boundary of the source plane (yellow rectangle) in (**b**) is the anisotropy index, above which in (**a**) the image is experiencing excessive perspective distortion and cannot be reliably transformed. The mathematical intuition of this function is shown in Supplementary Fig. S5.

**Figure 6:**
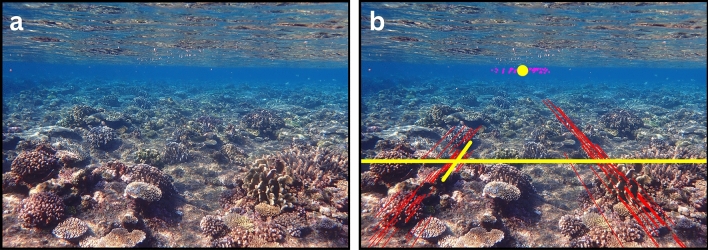
Two images demonstrate the effect of the Detect-Perspective-Grid function, as shown in Fig. 2. (**a**) The rotated image before detecting the perspective grid and (**b**) after detecting the perspective grid. Note the following features in (**b**): (i) the detected left and right candidate-vanishing lines are depicted as red lines, (ii) the candidate-vanishing points are depicted as pink dots, (iii) the overall-vanishing point is depicted as a yellow circle, and (iv) the perspective grid is depicted as yellow lines where the diagonal yellow line is the highest-ranking vanishing line (i.e., has the minimum perpendicular distance from the overall-vanishing point). Note (1) that here only 20% of the total number of candidate-vanishing lines are shown, which have the shortest perpendicular distances from the overall-vanishing point, and (2) that only a random sample of 1.25% of the total number of candidate-vanishing points are shown. These visualization-downscaling procedures were needed so that the hundreds to thousands of these features for any given image are discernable. All the candidate-vanishing lines and candidate-vanishing points for this image are shown in Supplementary Fig. S6, as well as the intermediate-image manipulations.

**Figure 7:**
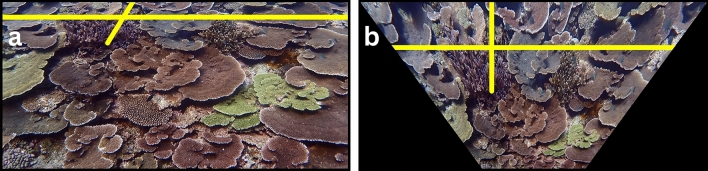
Two images demonstrate the effect of the Brute-Force Inverse-Perspective-Mapping function, as shown in Fig. 2. (**a**) Before brute-force inverse-perspective mapping and (**b**) after brute-force inverse-perspective mapping. The source plane in (**a**) has been extracted from the rotated image (i.e., all the pixels above the anisotropy index were cropped out) with the perspective grid drawn on the source plane shown here as yellow lines. The optimized top-down view is shown in (**b**). Note that the perspective grid depicted by yellow lines is at 90° in (**b**), indicating that the vanishing lines became parallel, and the perspective distortion has been removed. The intermediate-image manipulations are shown in Supplementary Fig. S7.

The image preprocessing (Fig. 2: Calibrate Camera Lens and Remove Lens Distortion; Supplementary Fig. S1), detecting and characterizing the scene geometry (Fig. 2: Detect Horizon, Remove Camera Roll, Detect Source Plane, and Detect Perspective Grid; Figs. 3, 4, 5, 6; Supplementary Figs. S3–S6), and searching for the transformation homography (Fig. 2: Brute-Force Inverse-Perspective Mapping; Fig. 7; Supplementary Fig. S7) showed successful transformation of reefscape images into top-down views (Fig. 7; Supplementary Figs. S9, S10; Supplementary Animation).

Calibrating the Olympus Tough TG-6 camera lens allowed us to remove the camera-lens distortions in the reefscape images, providing *ReScape* with images that were more representative of reality (Supplementary Fig. S1). A more-readily apparent effect of these well-established preprocessing functions is available in Supplementary Fig. S2. Detecting each scene’s horizon line (Fig. 3; Supplementary Fig. S3) allowed us to reliably remove the camera-roll angle (Fig. 4; Supplementary Fig. S4), define the search parameters of the source plane (Fig. 5; Supplementary Fig. S5), and find the most accurate vanishing line (Fig. 6; Supplementary Fig. S6). The camera-pitch angle, at which the images were taken relative to the reef, determined how much of each original image was preserved (Supplementary Figs. S9-S10), discarding excessively-distorted organisms toward the top of each image because they were not transformable (Fig. 5; Supplementary Fig. S5).

For each reefscape image, the vanishing-point detection visually matched the true vanishing point, allowing for the formation of an axis-aligned perspective grid that accurately characterized the perspective geometry of the scene (Fig. 6; Supplementary Fig. S6). Searching for the transformation homography via brute-force inverse-perspective mapping reliably converged each perspective-grid angle to 90° to remove the perspective distortion in each reefscape image (Fig. 7; Supplementary Figs. S9, S10). The *ReScape* algorithm achieved an 85% process rate (106/125 images) and a 95% success rate (101/106 images). Excluding camera-lens calibration, the 125 reefscape images were multi-processed by *ReScape* in 12.5 minutes (i.e., 10 images per minute on a computer equipped with an Intel^®^ Xeon^®^ E5-2660 v3 central-processing unit @ 2.60 GHz, 64 GB of random-access memory, and an NVIDIA^®^ Quadro^®^ K2000 graphics-processing unit) while averaging 35% random-access-memory utilization. There were seven distinct reasons why the 19 reefscape images aborted while being processed by the *ReScape* algorithm and representative cases for each aborted image are provided in Supplementary Fig. S12 with brief descriptions. *ReScape* removed all the perspective distortion in the x- and y-direction of the landscape images (Supplementary Fig. S8). However, very minor amounts of a different type of distortion were introduced in the y-direction toward the top of the image (Supplementary Fig. S8).

## Discussion

There have been, to our knowledge, only two attempts in the literature to quantify reef conditions from reefscape images^53,54^. Because perspective distortion prevented the quantification of the size and percentage cover of individual coral colonies, Haas et al.^53^ computed a single composite metric for each reefscape image to characterize its general aesthetic value, which was loosely interpreted as reef condition. For the same reason, Roberts et al.^54^ computed the presence and absence of different habitats in reefscape images, which was also loosely interpreted as reef condition. The present study therefore is the first to make reefscape images readily available for quantitative analysis of reef conditions. We achieved this feat by creating the *ReScape* algorithm to remove the perspective distortion from reefscape images by transforming the images into top-down views. Percentage cover data of individual coral colonies can now be extracted from transformed coral-reefscape images by techniques such as manual point-count analysis^55,56^, semi- or fully-automated point-count analysis^57–61^, or even semi-automated machine-learning classification of every pixel in the image^62^.

To consider the steps taken toward the successful transformation of a reefscape image, we first needed a technique to transform the perspective of the scene that removed distortions and displayed all marine taxa as their actual size. Such a transformation was in part already solved by the autonomous-driving industry because the distortionless, accurate real-time mapping of the surrounding environment was required to maximize passenger safety^24,25^. Inverse-perspective mapping relied on knowing the camera’s position and orientation (i.e., pose)^27^ to determine the homography for the transformation. Because autonomous vehicles have the camera mounted on the car at a known pose, the homography for the transformation can be directly derived from a set of equations^27,29^. For reefscape imagery, however, the photographer’s camera could be located anywhere in the water column and could be facing any direction. Therefore, we developed the *ReScape* algorithm to find the perspective-transformation homographies without knowing the camera poses.

To overcome the problem of unknown camera poses, the critical component of dynamically finding the homography for each reefscape image was locating the vanishing point. For autonomous vehicles, the vanishing point for each forward-facing road image can be detected based on the convergence of road-lane markings because they function as vanishing lines^63,64^. On coral reefs, however, there are no consistently occurring markings that function as vanishing lines. Therefore, to isolate candidate-vanishing lines, we applied various logical filters to all the detected lines from the edges of corals and other objects in the reefscape images. For each reefscape image, we averaged the intersections of the candidate-vanishing lines to find the overall-vanishing point and then applied various rankings to isolate the most representative-vanishing line. We used this representative-vanishing line in each reefscape image to form an axis-aligned perspective grid to find the optimal top-down view, which was evident when the perspective grid converged to 90° during the homography search. This approach allowed us to find the optimal top-down view of each reefscape image without knowing the camera pose.

Transforming reefscape images into top-down views enables ecologists to revisit archived reefscape images to quantify historical reef conditions and fill geographical gaps in our understanding of coral-reef dynamics. Historical reefscape images however may be biased toward the most aesthetically pleasing areas of reefs and not systematically collected using any form of randomization^23^. Therefore, regional generalizations based on only a few historical images should be treated with caution. We do nonetheless encourage studies to revisit archived reefscape images, especially before the first global coral-bleaching event in 1997/98, which caused extensive damage to coral reefs worldwide. Coral databases have minimal data before this period^7,8^. Coral-reef localities where data have remained sparse are some isolated reefs in the Pacific and Indian Oceans, reefs in India, and the Bay of Campeche in Mexico^7,8^. If the exact geographical locations of historical-reefscape images are not available in the image metadata, the georeferencing information can be provided by the photographer’s records, which will likely be at the regional, island, or dive-site level. For historical-reefscape images that were captured with a model of camera lens that may no longer be available, a different camera model with the same or comparable lens specifications and image dimensions can be used for calibration instead. Because the *ReScape* App dynamically computes and passes the camera-lens-calibration parameters to the remove-lens-distortion function, the lens of any standard camera can be calibrated and used to collect reefscape images.

The *ReScape* algorithm enables ecologists to leverage reefscape imaging as a new survey method for analyzing reef conditions and will likely be especially useful for rapid monitoring in response to disturbance events. The current industry standard for image-based surveys is the digital photoquadrat. Photoquadrats capture images from a top-down view—requiring that the camera is oriented perpendicular to the benthos. This approach forces the image plane to be equidistant from the camera throughout the entire photo—ensuring that perspective-related distortions are avoided and thereby readily enabling accurate estimates of coral-colony sizes and cover. However, the photoquadrats only capture a small area of the reef (≤ 1 m^2^)^20,65^, requiring many images to accurately characterize the coral assemblages^66,67^.

Recent advances in structure-from-motion technology^68^ produce three-dimensional (3D) models that have greatly increased the area of the reef being surveyed, provide valuable information about the 3D nature of reefs, and can be orthorectified into two-dimensional photomosaics for quantitative analysis of their top-down view^69,70^. However, generating and analyzing 3D models requires (i) hundreds to thousands of images at any given site, which is time-consuming, (ii) the purchase of expensive software and computational resources, (iii) substantial staff and training, and (iv) significant processing times^71^. While such limitations have restricted the structure-from-motion technique to high-budget projects, Sauder et al.^72^ recently overcame most of these limitations by adapting a machine-learning model^73,74^ to construct a fully classified 3D model of the reef in real-time by predicting the camera poses and taxonomic labels of every pixel from video feeds. While the breakthrough provided by Sauder et al.^72^ will likely increase the use of the structure-from-motion technique, the resolution of the 3D models is considerably reduced relative to those generated by proprietary software (e.g., Agisoft Metashape). Here, we add to the large-area imaging (sensu Ref.^75^) toolbox by creating the *ReScape* algorithm to enable the use of reefscape imaging as a rapid, cost-efficient survey method.

**Figure 8:**
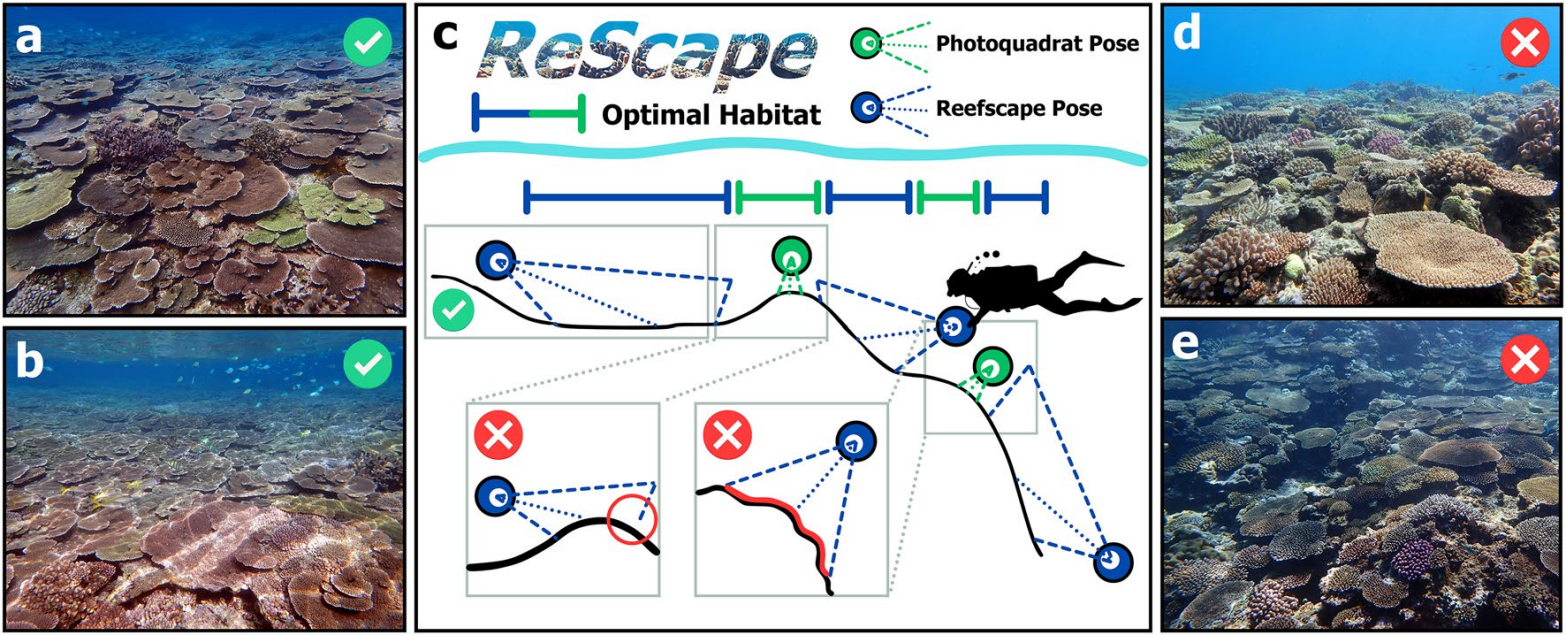
Best-practice surveying techniques to gain quantitative access to reef conditions in coral-reefscape images. The black-curved line in the central image represents a typical side profile of reef-habitat zonation. The camera poses of reefscape images, which are compatible with *ReScape* are blue circles and projection lines, which capture relatively large expanses of low to moderate rugosity reefs (**a**–**c**). Capturing many photoquadrats (green circles and projection lines) remains the most effective imaging technique for surveying along habitat boundaries because their small field-of-view ensures that the imaged reef is equidistant from the camera lens throughout the entire photo despite the high rugosity (**c**). Note in (**c**) that each reefscape pose has a blue-dashed line that is perpendicular to the optical axis (blue-dotted line), beyond which the camera does not receive light, forming the required horizon line where this perpendicular line meets the reef. Additionally, note in (**d**) an incompatible reefscape pose because the reef slopes downwards rather than vanishing [as also depicted by the red circle in the left-hand box in (c)]. Moreover, note in (**e**) an incompatible reefscape pose because (1) the field-of-view does not include the required horizon line and (2) the imaged reef has high rugosity [as also depicted by the red line in the right-hand box in (c)]. Also, note that all these fields-of-view are not to scale relative to the reef and instead demonstrate the optimal habitats for each imaging technique in relation to the reef rugosity.

Obtaining the scale and total-transformed area of reef captured by historical-reefscape images is difficult because these ‘recreational’ images rarely, if ever, contain scale bars. Based on the typical sizes of coral colonies, we estimate that the real-world length of the bottom border of the source plane in Fig. 7a is 3 m, corresponding to a transformed-imaged area of 35 m^2^ in Fig. 7b (i.e., using the area of a trapezoid equation). To assess the validity of our expert-derived estimate, we also obtained a scale for the image in Fig. 7b by dividing the average Euclidean pixel distance between the annual growth bands that are on the *Acropora hyacinthus* colony (Supplementary Fig. S11) by its average linear-radial-extension rate of 7 cm per year^76,77^, corresponding to a total-transformed area of 42 m^2^. For historical-reefscape images that do not have species with annual-growth bands, we argue that expert-derived estimation of scale is a reasonable approach because these estimates of the total-transformed area (i.e., 35 m^2^ and 42 m^2^) are similar. The expert-derived surveyed area of 35 m^2^, captured by a single-reefscape image, would have required 35 large-size (i.e., 1 m^2^) photoquadrats, conservatively translating to a 90% reduction in imaging effort (Supplementary Fig. S11). This improvement in the efficiency of underwater coral-reef survey methodology using monocular-image replicates, owing to the very different ways that photoquadrat and reefscape images are composed (Fig. 8), rivals when photoquadrats replaced labor-intensive underwater methods in the 1990s. However, because the exact scale of historical-reefscape images cannot be rigorously determined and the percentage coral cover is scale invariant, coral cover is the most suitable metric for analyzing historical-reefscape images using our method. Moreover, other computer-vision techniques can account for variation in the relative scale between historical-reefscape images that have been transformed by *ReScape* by matching visual-texture patterns at multiple scales^78^. Using the *ReScape* algorithm, ecologists are equipped to efficiently survey reefs with reefscape imaging, increasing the spatio-temporal extent and resolution of future survey designs. Reefscape imaging is no longer strictly a recreational-imaging technique.

While the *ReScape* algorithm is an advancement for underwater coral-reef survey methods, not all reefscape images are compatible with the current version of *ReScape.* Therefore, several assumptions and limitations need further consideration. Because inverse-perspective mapping assumes that the source plane is flat, minor distortions are introduced, particularly around the top of the image because it covers more area of an imperfectly-flat reef (Fig. 7b; Supplementary Figs. S8-S10). While these new distortions persisted in autonomous-driving applications for nearly three decades, Bruls et al.^44^ greatly reduced these new distortions by training a machine-learning model to learn the transformation homography for imperfectly-flat surfaces by iteratively updating a composite top-down-view image from sequentially transformed frames of road scenes from forward-facing video feeds.

Because the horizon line is the basis of the extraction of perspective geometry, the horizon line of the reef must be visible and intersect the left side of the image (i.e., the y-axis). In other words, the image must be composed of mostly reef and some water column, ideally 85% reef and 15% water column. If the horizon line is too high in the image, then the improved visibility in the background causes the demarcation of the edge-pixel density to be less pronounced, resulting in a reduced accuracy for the detection of the horizon line, which in an extreme case was responsible for 1 of the 19 processing-failure cases (Supplementary Fig. S12). This horizon-detection inaccuracy was slightly below the discernable horizon line, which resulted in a detected perspective-grid angle that was also too low. This angle problem stemmed from the biased-low y-intercept of the horizon line filtering for line intersections that were not positioned on the true-horizon line, which resulted in the selection of the highest-ranking vanishing line that had a shallow angle (i.e., low bias). This low-biased perspective-grid angle thereby misrepresented the perspective geometry of the scene. By beginning the homography search with a biased-low perspective-grid angle, the brute-force inverse-perspective mapping ‘over-corrected’ the transformation during the homography search because the angle took considerable time to converge to 90°, which reversed the direction of perspective distortion along the y-axis of the image. Inversely, suppose the horizon line is too low in the image, then the reduced visibility of the entire reef plane causes more rapid perspective distortion, resulting in a smaller source plane and less-transformed reef area. Again, we found that a reefscape image composition of 85% reef and 15% water column provides an optimal trade-off between the accuracy of the horizon line and the amount of transformed area.

High turbidity scenes are also not particularly compatible with the *ReScape* algorithm because the particles in the water column interfere with Canny-edge detection—a critical component of detecting the horizon, source plane, and perspective grid—preventing the characterization of the scene’s perspective geometry. In theory, turbid reefscape images could become compatible with the *ReScape* algorithm if future work were to (i) incorporate the sophisticated color-correction method provided by Akkaynak & Treibitz^79^ as an additional preprocessing function, and (ii) use red, green, and blue, and image-depth (D) imaging, because information on the distance of each pixel from the camera lens and the depth of the camera in the water column are required to parameterize such a physics-based method. The use of archived, turbid-reefscape images without a D channel would require future work to extend image-depth estimation methods (e.g., Ref.^80^) to work without any a priori water-depth information. A similar reduction in the performance of *ReScape* was observed for especially low-resolution reefscape images with less than 0.75 megapixels. This minimum-resolution threshold was obtained by iteratively decreasing the resolution of our historical reefscape images, which were 12 megapixels. While low resolution is not a concern for full-, high-, and ultra-high-definition modern images, reefscape images that were captured before the commercial release of full-definition technology in 1991 can be resized. Additionally, film photographs and slides can be scanned into a digital format and processed by *ReScape*.

In addition to the minor horizon-detection inaccuracy for images with a horizon line too high in the image and the turbidity-induced processing errors, inverse-perspective mapping assumes that the reef is planar, and therefore only scenes with relatively low to moderate rugosity can be reliably transformed (Fig. 8). We noticed that sections of the reefscape images that violated the low-rugosity assumption (i.e., large crevices, steep slopes, and anomalously vertical coral colonies) became stretched after the perspective transformation. Considering these assumptions and current limitations, we recommend that future reefscape image surveys adhere to the following rules (Fig. 8) to increase the performance of the *ReScape* algorithm:

1. Find a relatively low to moderate rugosity area of the reef that vanishes toward the background (Fig. 8a,b).
2. Avoid reefs with extremely high turbidity or otherwise low visibility (e.g., deep depths and low daylight).
3. Compose the minimally-occluded horizon line approximately one-eighth of the height from the top of the image frame (Fig. 8a,b).

Reefs that simply slope downward and away from the photographer may appear to resemble a horizon line (Fig. 8d), however, they do not qualify as a horizon line by definition; the reef must vanish toward the background of the image (Fig. 8a,b). While the incompatible image in Fig. 8d will likely appear to be successfully processed, it will slightly overcorrect during the Brute-Force Inverse-Perspective-Mapping function. Because these image-compatibility rules were developed on historical coral-reefscape images, by evaluating which of the 125 testing images were successfully and unsuccessfully transformed, the images we used are considered equivalent to those captured by recreational photographers. Indeed, the first coral-reefscape image captured while knowing the optimal-imaging rules has yet to be taken. Therefore, coral-reefscape images collected in the future, while closely following our imaging rules, should observe even better processing rates. The scale of reefscape images collected in the future can be accurately determined by placing a scale bar parallel to the reef plane, allowing for the use of all conventional metrics for analyzing reef conditions. The performance of the *ReScape* algorithm can be further improved by avoiding the more nuanced image-composition complications that are described in Supplementary Fig. S12. Additionally, because the *ReScape* algorithm is incompatible with capturing the reefscape image across multiple habitats, digital photoquadrats remain the most effective imaging technique to survey coral assemblages across habitat transitions (Fig. 8). While we developed the *ReScape* algorithm for reefscape images, we did not use any inherently unique visual characteristics of coral reefs when developing the *ReScape* algorithm, and therefore *ReScape* can be used to assess other marine, and even terrestrial, ecosystems.

We successfully present the first-ever ecological application and extension of inverse-perspective mapping to transform the perspective of underwater reefscape images into top-down views. This transformation was needed because perspective distortion causes coral colonies close to the camera lens to appear disproportionately larger than coral colonies far from the camera lens. To rectify this century-long distortion problem we developed the *ReScape* algorithm to overcome the issue of unknown camera locations and orientations for inverse-perspective mapping. The *ReScape* algorithm unlocks innumerable reefscape images, many taken by citizen scientists and recreational photographers, that can now be transformed into top-down views to examine the historical conditions, dynamics, and trajectories of coral reefs, worldwide. The *ReScape* algorithm also enables the use of reefscape imaging to design more ambitious coral-reef surveys in the future.

Machine learning and other computer-vision tools promise to expedite image-based surveys and data extraction. However, these valuable tools are typically provided as numerous scripts of Python code which can be difficult to use because (i) coding, especially in Python, is not a universal skill among ecologists, and (ii) the use of the code requires installation of Python, an interactive development environment, and software dependencies. These prerequisites add complexities that can reduce the number of users. Therefore, here we provide *ReScape* as a readily-accessible, user-friendly App that automatically compiles all the required software and dependencies, allowing easy use for anyone with a computer and internet access even without any coding experience.

In conclusion, by opening a new era of coral-reef monitoring, our *ReScape* algorithm (i) increases the efficiency of surveying coral reefs, (ii) aims to further engage citizen science in coral-reef research, (iii) responds to the call by Carey et al.^81^ for more interdisciplinary science among ecologists and computer scientists that are producing transformative innovations, which are accelerating inquiry, analysis, and understanding of the world’s coral reefs, and (iv) provides a readily-accessible method to increase historical and geographical information to help sustain coral reefs in the face of rapid climate change.

### Supplementary Information

Supplementary Video 1.

Supplementary Information.

## Data Availability

The *ReScape* App and the source code for the *ReScape* algorithm (hereafter ‘*ReScape*’) will both be made available upon publication at the *ReScape* GitHub repository: https://github.com/InstituteForGlobalEcology/ReScape. *ReScape* is licensed under Creative Commons Attribution-NonCommercial-ShareAlike (CC BY-NC-SA) 4.0 International. The authors elected the CC BY-NC-SA licensing to (i) promote controlled, readily accessible, and free use of *ReScape* among non-commercial parties primarily in the academic, research, and education sectors and (ii) allow others to contribute to the continued development of *ReScape*. Notably, the CC BY-NC-SA license requires any use of *ReScape* to (i) attribute the authors, (ii) be non-commercial, and (iii) inherit the CC BY-NC-SA license in perpetuity if derivative works are created. These legally-binding requirements are non-exhaustive and therefore the full CC BY-NC-SA legal code is available at the *ReScape* GitHub repository in the LICENSE.txt file. Those who wish to use *ReScape* in any commercial capacity must inquire with the corresponding author for arrangements, with the following notable exceptions: the authors personally guarantee that we will not enforce our reserved rights for unnotified use that could be perceived as restricted by (i) for-profit academic publishers who may disseminate manuscripts that reference, implement, or extend *ReScape* and (ii) for-profit media channels who may feature *ReScape* in press releases (i.e., these are the only usages of *ReScape* that could be perceived as restricted that we permit without notification unless correspondence with the authors determines otherwise). Those who wish to create derivatives of *ReScape* are encouraged to inquire with the corresponding author for collaboration but are by no means required to do so. This Data Availability statement is not legal advice and is only a guiding summary of how *ReScape* can (and is expected to) be used, and we, therefore, reserve the right to enforce the terms of the CC BY-NC-SA license at our discretion at any time, provided that any users of *ReScape* without an exception either (i) as described above or (ii) personally issued by the corresponding author should not adhere to the full legal code of the CC BY-NC-SA license. If users have any remaining questions regarding our legal interpretation of their intended use of *ReScape*, please inquire with the corresponding author. This release of *ReScape* is version one. If our users develop improvements to *ReScape* for their specific use cases, we encourage ‘pull’ requests to our GitHub repository so we can collaboratively make future versions of *ReScape* (i) compatible with increasingly complex image compositions and (ii) available for all ecologists, reef managers, citizen scientists, and recreational photographers.
