# Supplementary material for: ReScape: transforming coral-reefscape images for quantitative analysis
Source: Sci Rep. 2024 Apr 17;14:8915. doi: 10.1038/s41598-024-59123-2 (PMC11024090; doi:10.1038/s41598-024-59123-2)
Supplement: Supplementary file 2 — Supplementary Information. [file 41598_2024_59123_MOESM2_ESM.pdf]

Supplementary Information

ReScape: transforming coral-reefscape images for quantitative analysis

Z. Ferris, E. Ribeiro, T. Nagata, R. van Woesik

The only detection that was drawn on the images throughout the entire *ReScape* algorithm is the axis-aligned perspective grid, as it needed to be segmented during Brute-Force Inverse-Perspective Mapping. All other detections drawn on the images throughout this paper were purely for visualization purposes.

The intermediate steps for the Remove-Lens-Distortion function, as shown in Supplementary Fig. S1: (a) the raw reefscape image; (b) the image without lens distortion with calculated corner movement, marked with green dots, and the absence of the camera's field of view, the thin-black margins along the image borders; (c) y-axis-aligned corners calculated by using the standardized y-coordinates of the top and bottom pair of corners marked with green dots (this step produced a nearly indiscernible, but required change to ignore the absence of the camera's field-of-view); and (d) the extracted maximum, axis-aligned rectangle. Note that the dimensions of (d) are reduced owing to the narrower, axis-aligned field of view relative to (a). Also note that the *Pocillopora* sp. colony at the very bottom-left corner of the image in (a) is stretched toward the corner, which is corrected in (b). Additionally note that toward the top of the image in (a) the horizon line bows upward at the image borders, which is linearized in (b).

Intermediate Steps for **Remove Lens Distortion**

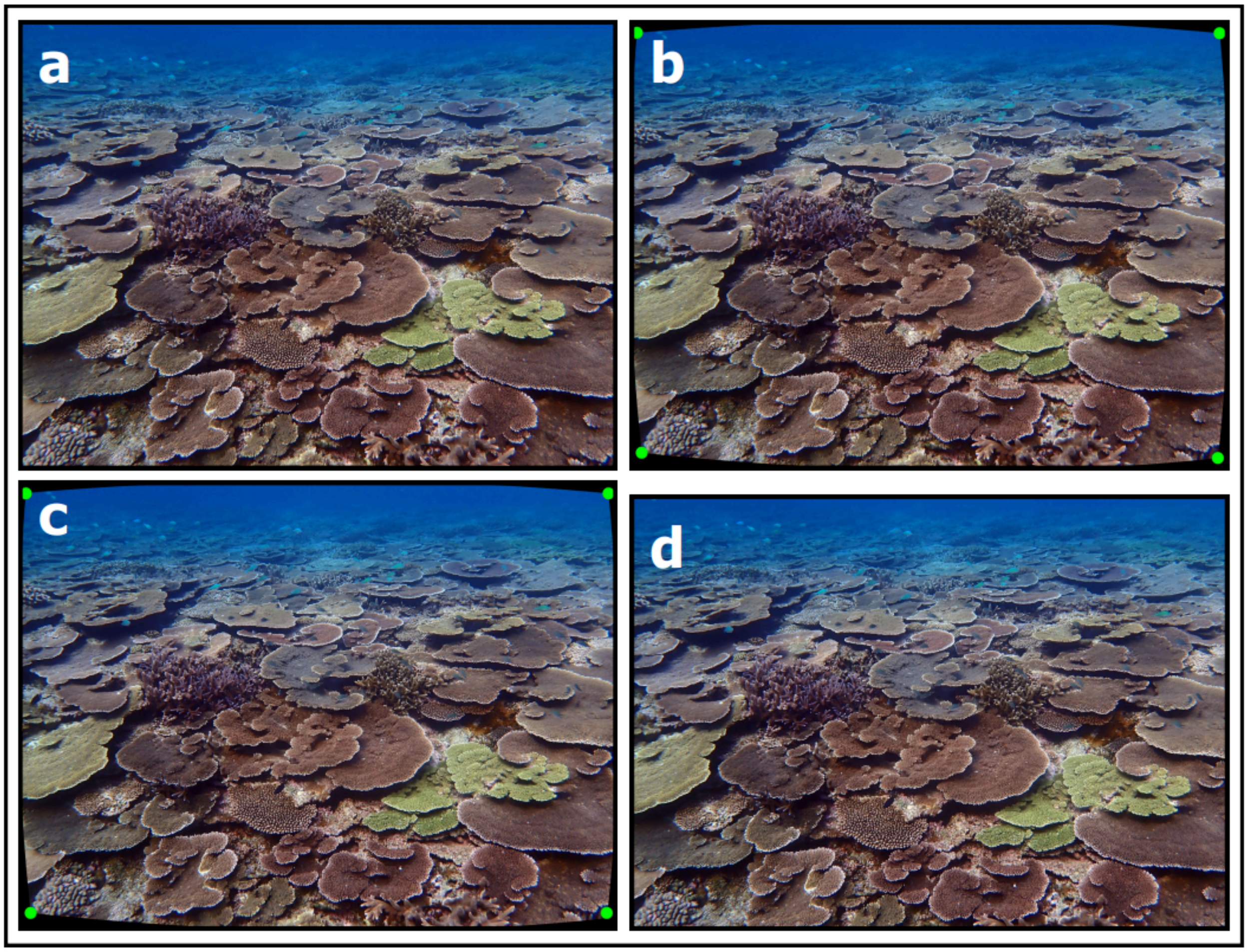

Supplementary Figure S1. Supporting visual for the Remove-Lens-Distortion function.

A More Readily Apparent Effect of the **Remove-Lens-Distortion** Function

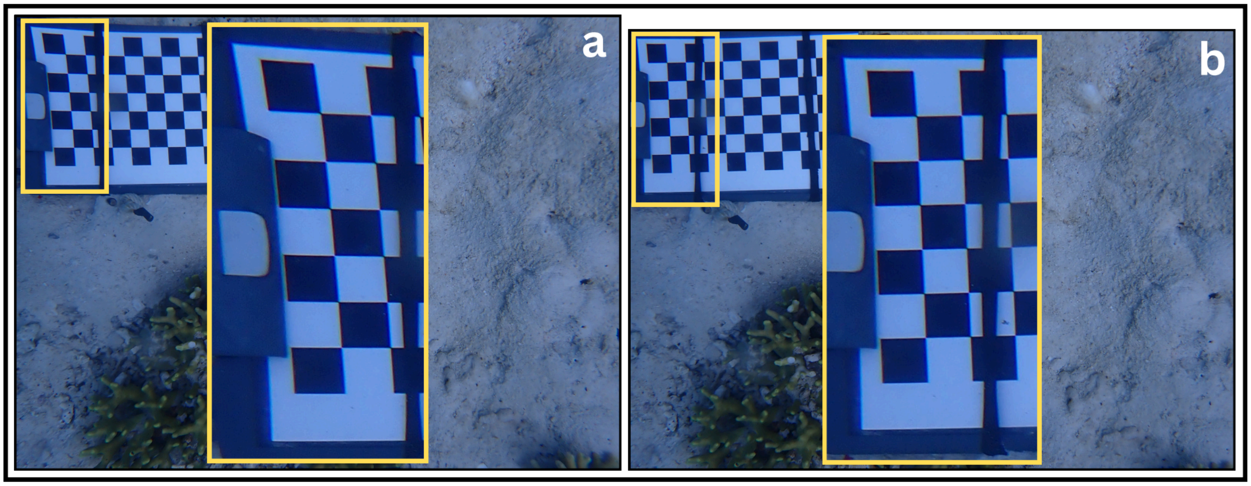

Supplementary Figure S2. Removing the lens distortion from an example camera-lens-calibration image to show the effect of the Remove-Lens-Distortion function, as depicted in Supplementary Fig. S1. (a) Lens-distorted image and (b) image without lens distortion. The yellow inset in each panel is zoomed in on the upper-left region of each image where the radial distortion was most substantial. Note that in (b) the squares and lines are no longer distorted. Here, panels (a) and (b) are equivalent stages of the Remove-Lens-Distortion function to panels (a) and (d) in Supplementary Fig. S1, respectively.

The intermediate steps for the Detect-Horizon function, as shown in Fig. 3, and in Supplementary Fig. S3: (a) the image without lens distortion; (b) the greyscale image with a 5 x 5 convolutional-kernel Gaussian blur; (c) the adaptive-thresholded image; (d) the edge-map produced by Canny-edge detection using a 5 x 5 Sobel operator; (e) the edge-density map produced by a 15 x 15 convolutional kernel, where the blue and red pixels have normalized edge-density values of 0 and 1, respectively; (f) the red line indicates the regressed water-column line in (e) using Theil-Sen robust-linear regression of the 100 randomly-sampled points from (e) that have edge-density values of zero; (g) ignoring pixels in (e) above the regressed water-column line in (f) by their reassignment to black; (h) the dashed-orange line indicates the optimal Otsu threshold and the panel insets indicate representative regions in (g) whose edge-density intensity-channel values are either lower or greater than the optimal Otsu threshold; (i) the Otsu-binarized image (i.e., pixels whose edge-density intensity-channel value exceeds the Otsu threshold were reassigned to white, and vice versa for the pixels who were reassigned to black); (j) the black points indicate all of the largest contour points from (i); (k) the blue line indicates the detected horizon in (a), which is obtained by regressing the filtered-contour points from (j) (i.e., removed those within 20 pixels from the border of the image and those whose y-coordinate had a z-score greater than 1.5); and (l) the white line indicates the detected horizon in (a), which is equivalent to the blue line in (k).

Intermediate Steps for **Detect Horizon**

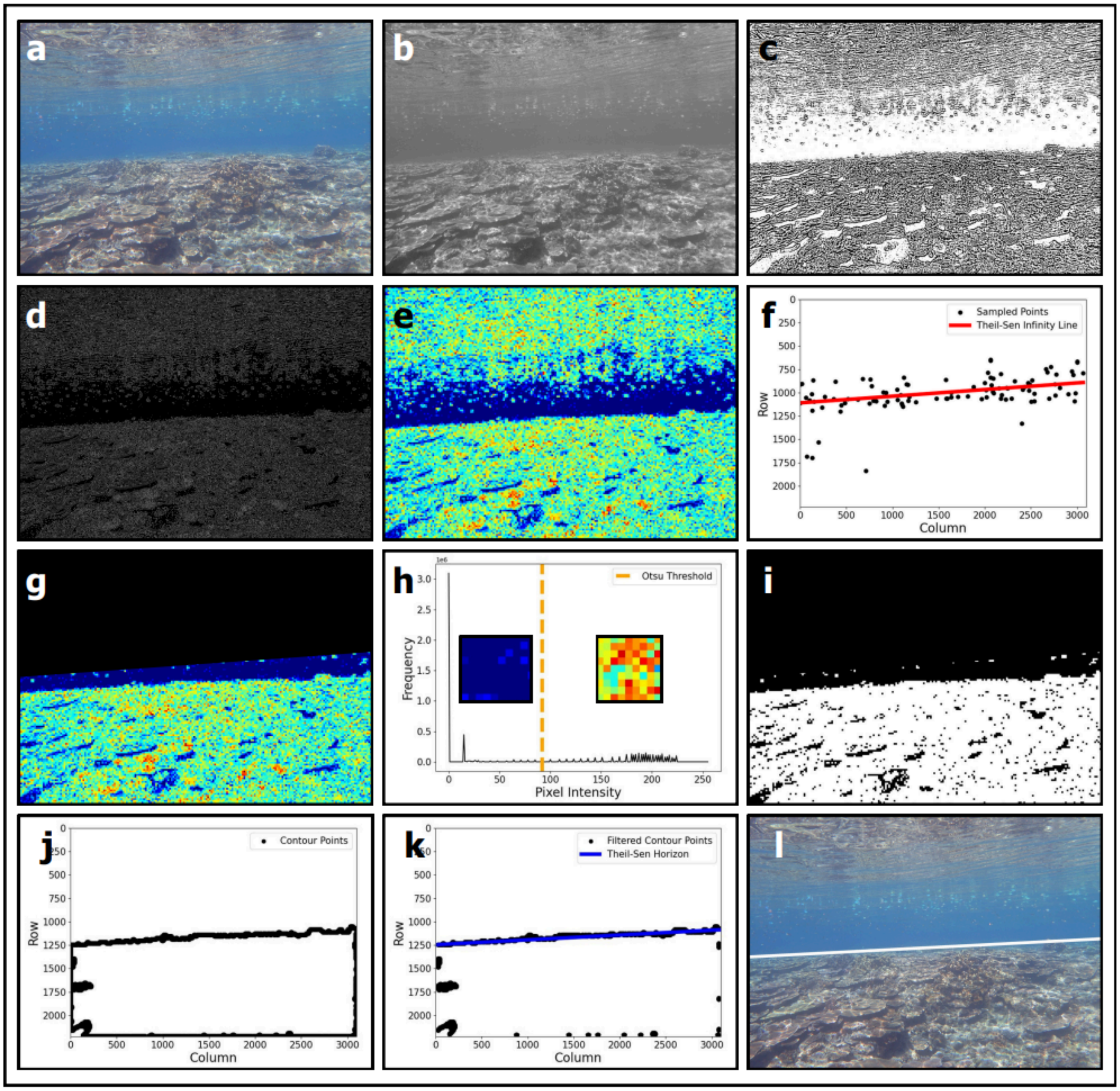

Supplementary Figure S3. Supporting visual for the Detect-Horizon function.

The intermediate steps for the Remove-Camera-Roll function, as shown in Fig. 4, and Supplementary Fig. S4: (a) the image without lens distortion; (b) the rotated image with the absence of the camera's field of view (i.e., the thin-black margins along the image borders); and (c) the extracted maximum, axis-aligned rectangle. Note that the dimensions of (c) are reduced relative to (a) owing to the narrower, axis-aligned field of view. Also, note in (c) that the white line depicting the horizon line is aligned to the x-axis, which makes the closer- and further pairs of destination corners equidistant from the camera lens, thereby reducing the destination-corner search to a one-dimensional problem.

The Intermediate Steps for **Remove Camera Roll**

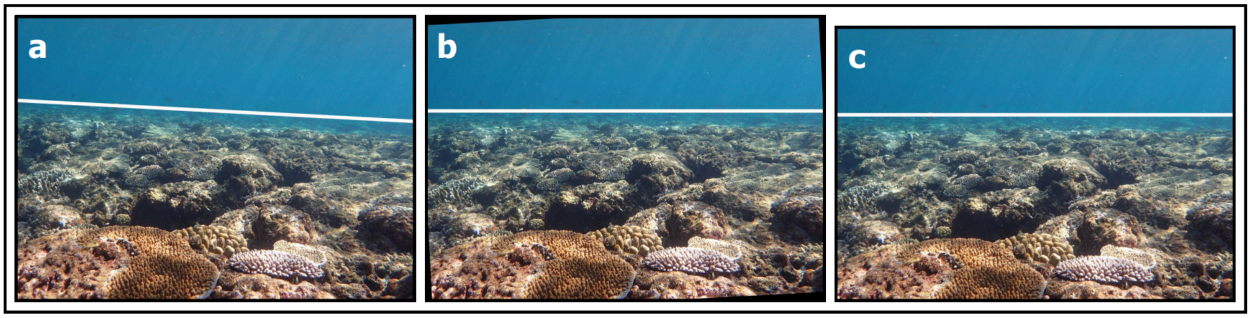

Supplementary Figure S4. Supporting visual for the Remove-Camera-Roll function.

The mathematical intuition of the Detect-Source-Plane function, as introduced in Fig. 5, and as shown in Supplementary Fig. S5: the edge map shown in (c) was generated by applying a Gaussian blur using a 5 x 5 convolutional kernel on the greyscale-rotated image, which was then further processed by the Canny-edge detector using a 3 x 3 Sobel operator. The anisotropy of the rotated edge map is computed within the moving window framework (i.e., the yellow rectangle), as shown in (c). The moving window (1) begins at the horizon (i.e., the top-orange line), (2) moves downward row-by-row, as indicated by the three yellow dots, and therefore, negates the need for filtering the edge map above the horizon line, which is required when detecting the perspective grid, as shown in Supplementary Fig. S6, and (3) stops halfway toward the bottom of the edge map (i.e., at the bottom orange line; i.e., not the midpoint of the vertical axis of the edge map, but the midpoint between the horizon and the bottom of the edge map and therefore, this stopping index is dynamically calculated for each edge map), as shown in (c). The red boxes [as shown in (c) and the zoomed-in view in (a)] highlight a region toward the background of the edge map that is very anisotropic (low-edge-orientation variance). Conversely, the blue boxes [as shown in (c) and the zoomed-in view in (b)] highlight a region toward the foreground of the edge map that is much less anisotropic than the texture depicted in (a) (i.e., has high-edge-orientation variance). Note that the anisotropy of the edge map starts at a maximum value at the horizon, and the anisotropy collapses to a minimum value while the moving window moves toward the stopping index [as shown in (c-d)]. The green line in (c) is the detected anisotropy index (i.e., where the camera is beginning to favor the detection of increasingly less anisotropic texture features toward the foreground of the edge map), which is used to define the y-coordinate of the top-source corners.

Mathematical Intuition for **Detect Source Plane**

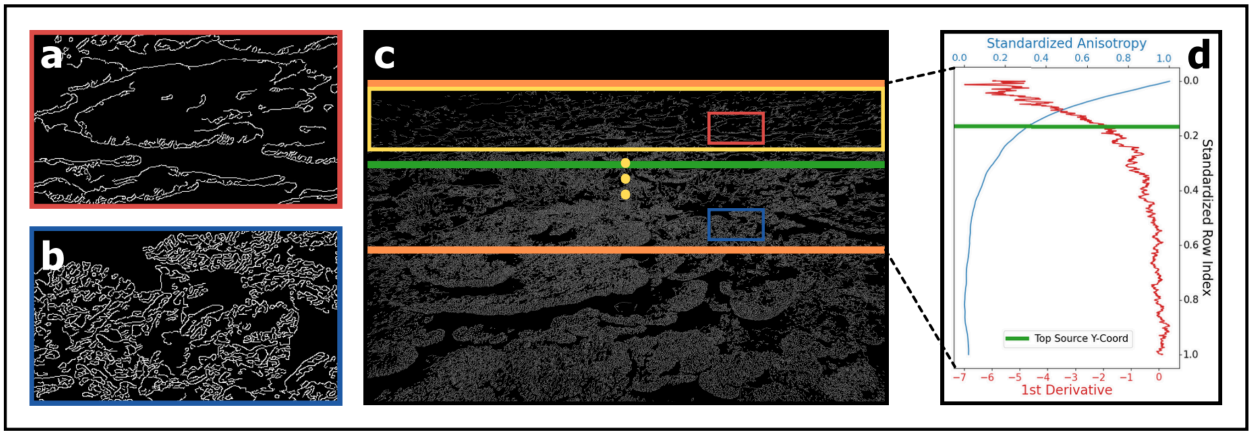

Supplementary Figure S5. Supporting visual for the Detect-Source-Plane function.

The intermediate steps for the Detect-Perspective-Grid function, as shown in Fig. 6, and in Supplementary Fig. S6: (a) the rotated image; (b) the greyscale image with a 5 x 5 convolutional-kernel Gaussian blur; (c) the edge map that is produced by Canny-edge detection using a 3 x 3 Sobel operator; (d) the filtered edge map (i.e., removing all of the edges above the horizon line); (e) all of the Hough-line detections (red lines) from the edges in (d); (f) the angle-filtered-grouped lines from (e) (i.e., candidate vanishing lines); (g) all of the intersections (pink dots) created by each unique pair of a left line and a right line in (f); (h) the candidate vanishing points are indicated by pink dots, which only include points from (g) that (1) lay within 25 pixels from the horizon line and (2) are within 25% of the image width centered on the midpoint of the image width, and the average vanishing point indicated by a yellow circle; and (i) the detected perspective grid indicated by yellow lines, where the diagonal line is the highest-ranking vanishing line which has the minimum perpendicular distance from the average vanishing point and whose y-coordinate midpoint is visually below the anisotropy index.

Intermediate Steps for **Detect Perspective Grid**

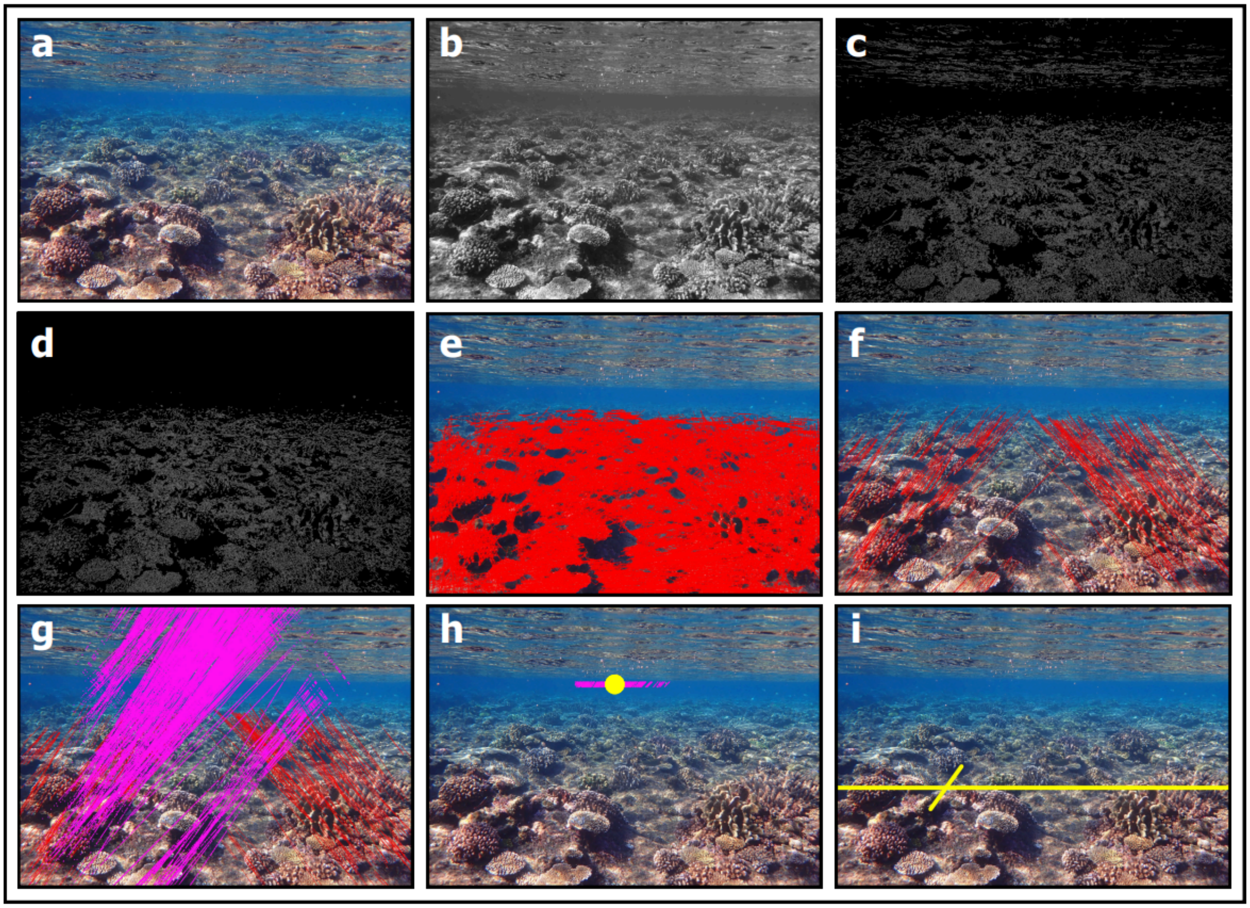

Supplementary Figure S6. Supporting visual for the Detect-Perspective-Grid function.

Intermediate steps for the Brute-Force-Inverse-Perspective-Mapping function, as introduced in Fig. 7, and as shown in Supplementary Fig. S7: (a) a randomly selected search iteration (x_{cf} = 0.630) obtained from the reefscape image presented in Fig. 7 with the perspective grid depicted as green lines whose blue, green, and red (BGR) values are 0, 255, and 0, respectively; (b) the segmented, closed mask of the perspective grid in (a) obtained by using (1) hue-, saturation-, and value-threshold (HSV) values of 60, 255, and 255, respectively, for the segmentation (i.e., each HSV-color channel's lower- and upper-threshold value is the same to isolate only the 60, 255, 255 HSV-color space of the perspective grid's 0, 255, 0 BGR-color space) and (2) a 5 x 5 closing kernel for the noise reduction, which is particularly helpful to improve the Canny-edge and Hough-line detection for decreasing values of x_{cf} when the perspective grid was experiencing increasingly more interpolation, which is zoomed in for visualization purposes; (c) the detected edges of the perspective grid's segmented mask in (b); (d) the detected Hough-lines from the perspective grid's edges in (c) are depicted in red where θ denotes the computed angle; and (e) the complete search results. Note that θ in (a-d) is less than 90°, indicating that continued compression (i.e., reducing x_{cf}) of the distance between the closest destination corners is required to converge θ to 90°. Also, note that the optimal compression factor here of 0.280 produced the optimal top-down view presented in Fig. 7b. The perspective grid is drawn as yellow lines in Figs. 6-7 and Supplementary Fig. S6 for visual contrast.

Intermediate Steps for **Brute-Force Inverse-Perspective Mapping**

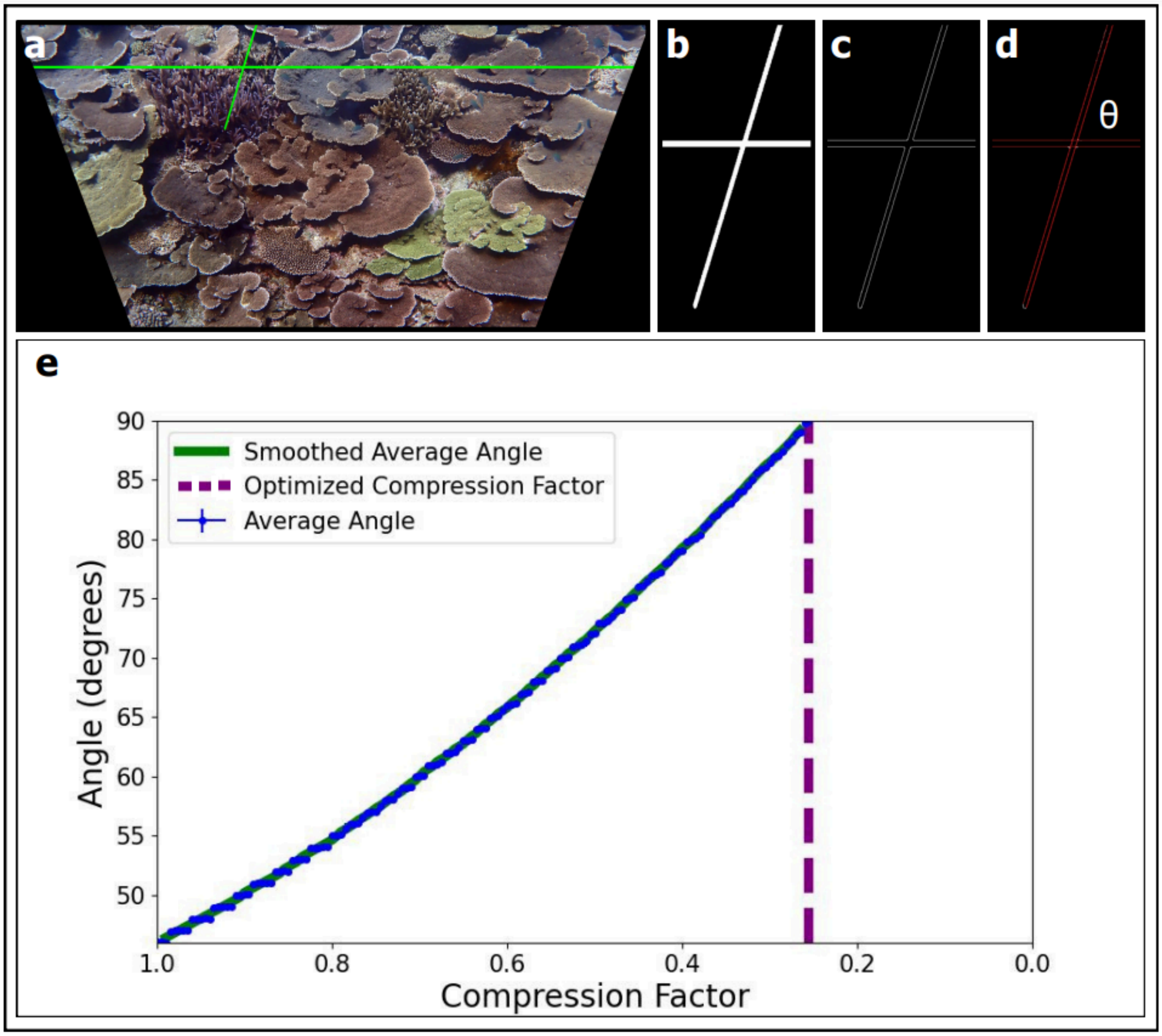

Supplementary Figure S7. Supporting visual for the Brute-Force-Inverse-Perspective-Mapping function.

Visual and quantitative validation of *ReScape*, as shown in Supplementary Fig. S8: the apparent length of same-length line segments decreases with increasing distance from the camera in two landscape images (c-d) because of perspective distortion, which has been corrected (a-b) in their transformed top-down views (e-f). Note that the transformed top-down views look the same as their ground-truthed top-down views (g-h). For each image (c-h), the pixel distance of each line segment was normalized to either the width (left-side panels) or height (right-side panels) of the image and plotted against the line-segment number (a-b), where segment 1 is closest to the camera in the landscape images (c-d) and the last segment is the furthest from the camera. The curves in (a-b) measure perspective distortion, whereas horizontal curves have zero perspective distortion. The perspective distortion was entirely removed in the x-direction (a). While the perspective distortion was also removed in the y-direction (b), new distortions were introduced because the land is an imperfectly flat surface, as seen by the slightly non-horizontal curve for the transformed image. The transformed images in (e) and (f) covered areas of 1,230 m^2 and 1,533 m^2, respectively. Note in the ground-truthed top-down views (g-h) that the goalie box was not painted as a rectangle.

Visual and Quantitative Validation of *ReScape*

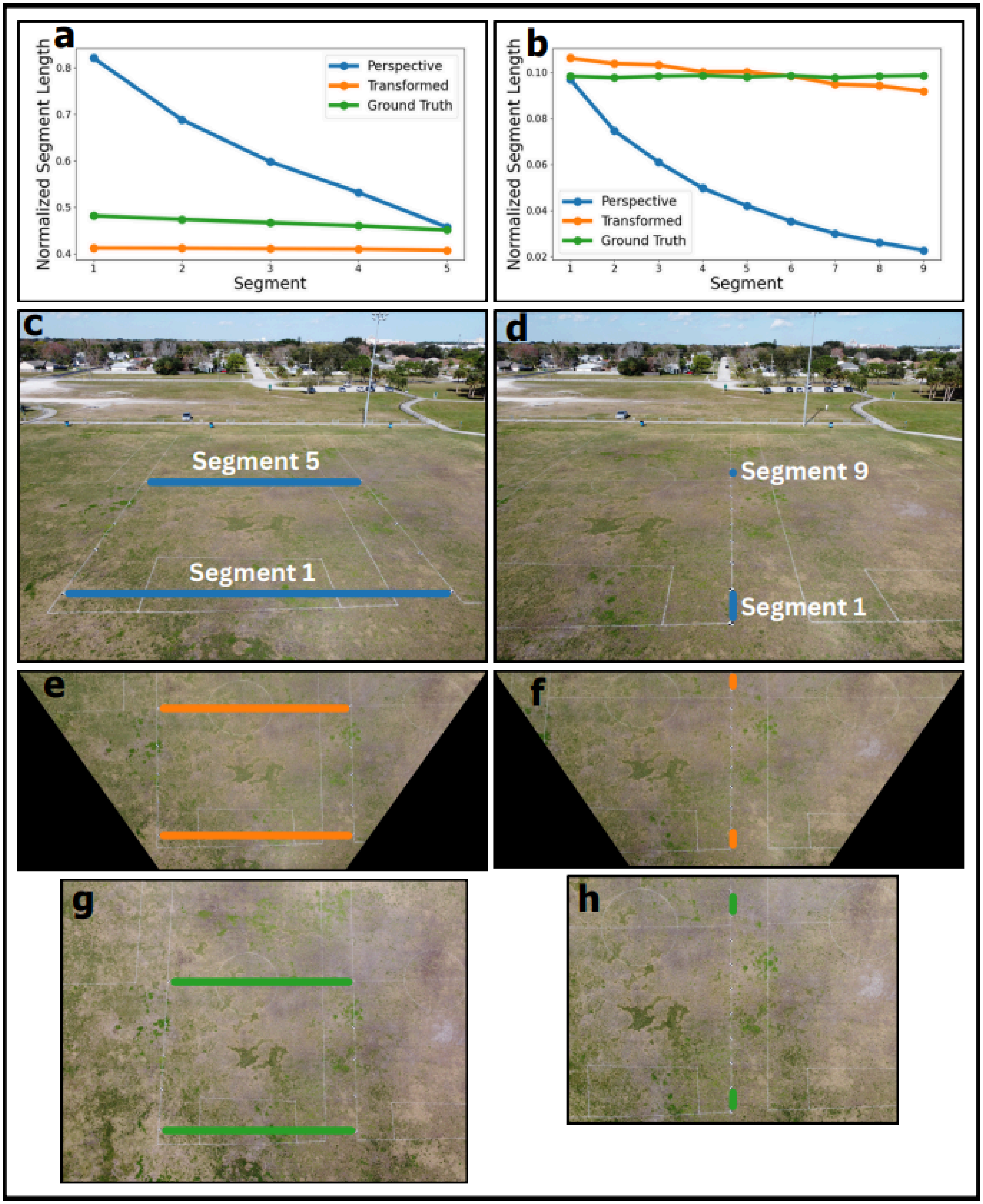

Supplementary Figure S8. Supporting visual for the visual and quantitative validation of *ReScape*.

ReScape's **Representative Success Cases**

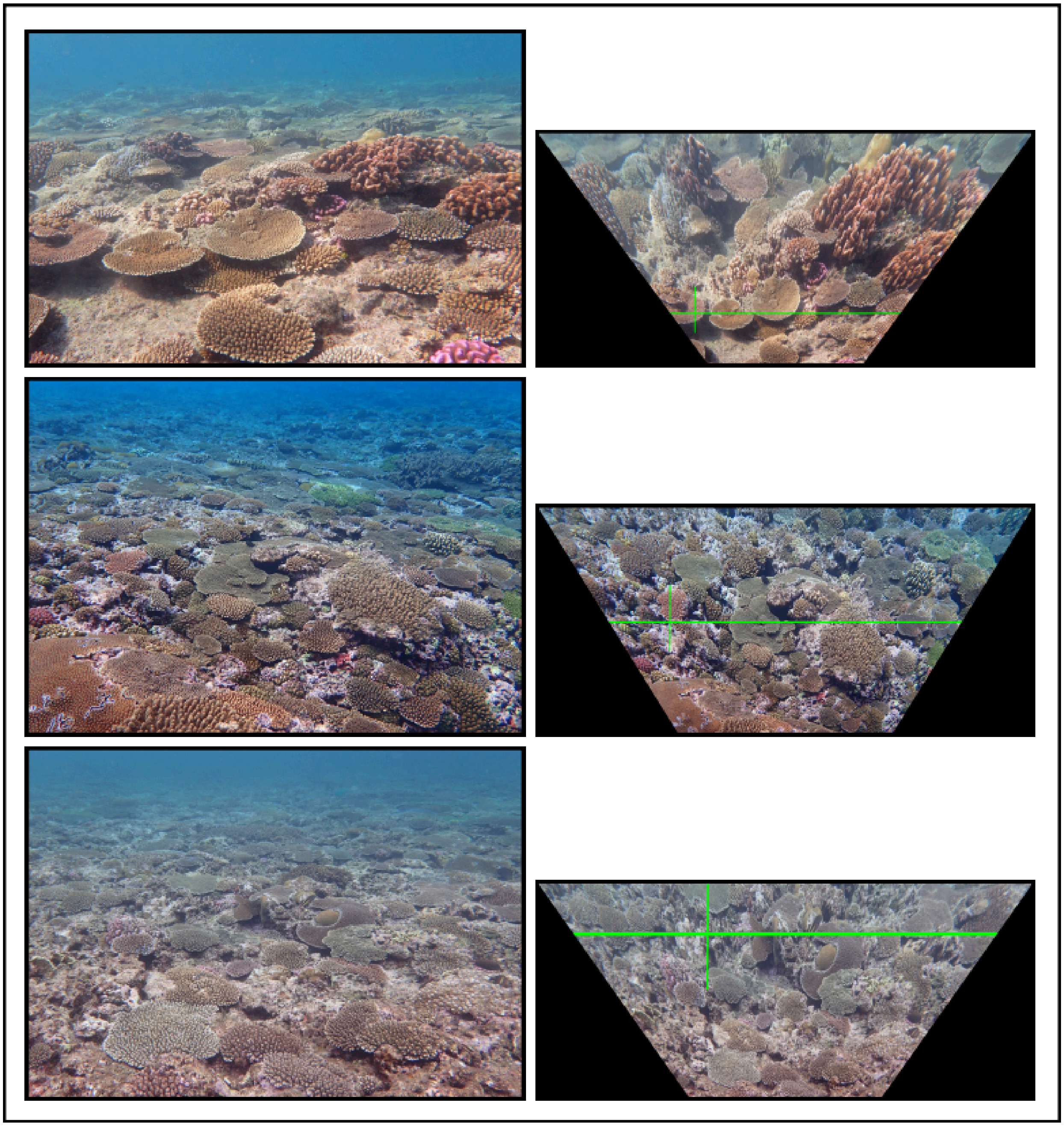

Supplementary Figure S9. Representative success cases produced by the *ReScape* algorithm. The rotated reefscape images (left) and the corresponding top-down views (right). The 'missing' upper region of each top-down-view is not included for the detection of the source plane, as the missing upper region is either not a reef (i.e., water column or water surface) or is an excessively anisotropic reef and, therefore, is not transformed.

ReScape's **Representative Success Cases**

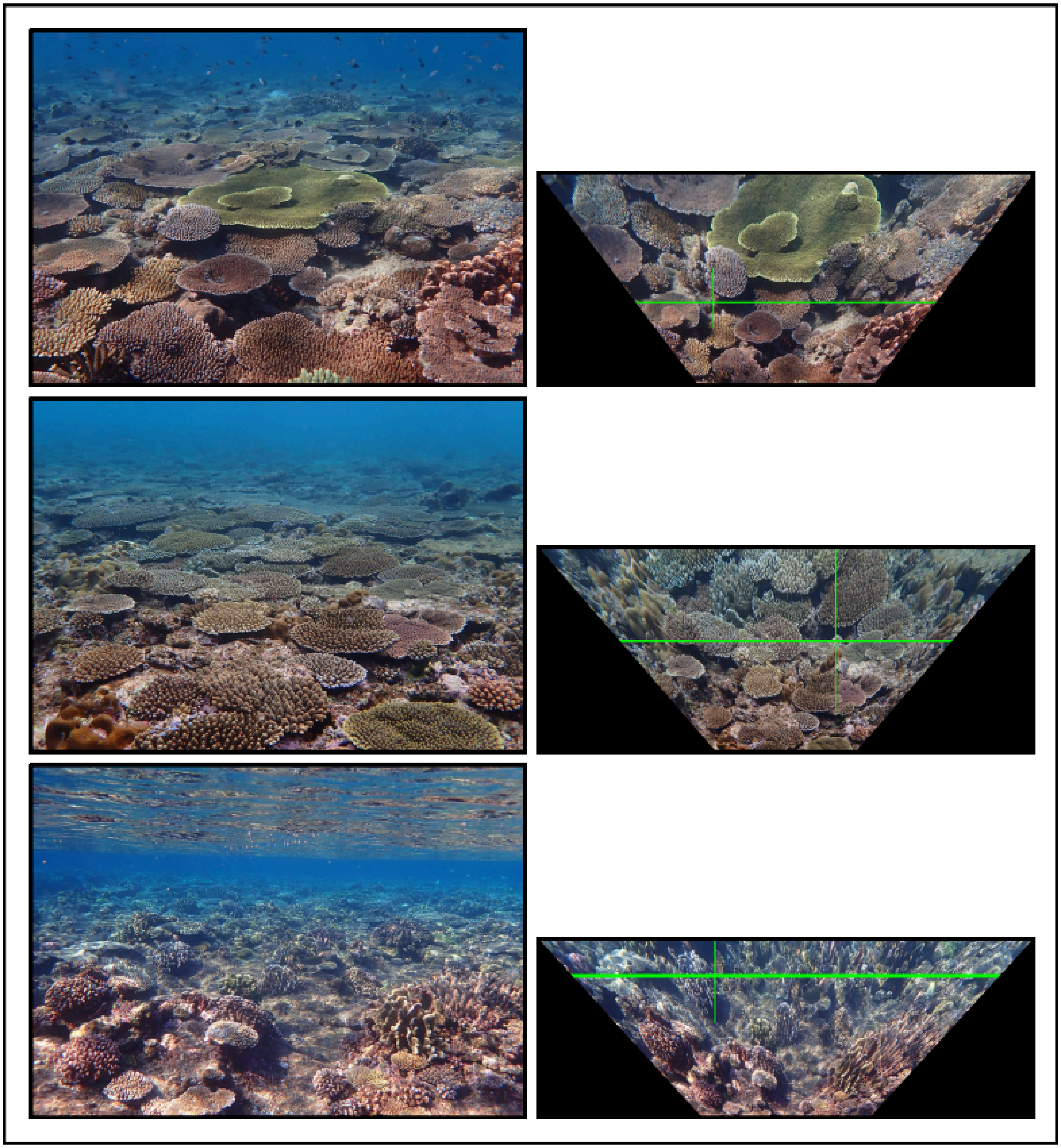

Supplementary Figure S10. More representative success cases are produced by the *ReScape* algorithm. The rotated images (left) and their corresponding top-down views (right). The 'missing' upper region of each top-down-view is not included for the detection of the source plane, as the missing region is either not a reef (i.e., water column or water surface) or is an excessively anisotropic reef and therefore is not transformed.

Expert-Estimated Scale of a Transformed Reefscape Image Relative to Photoquadrats

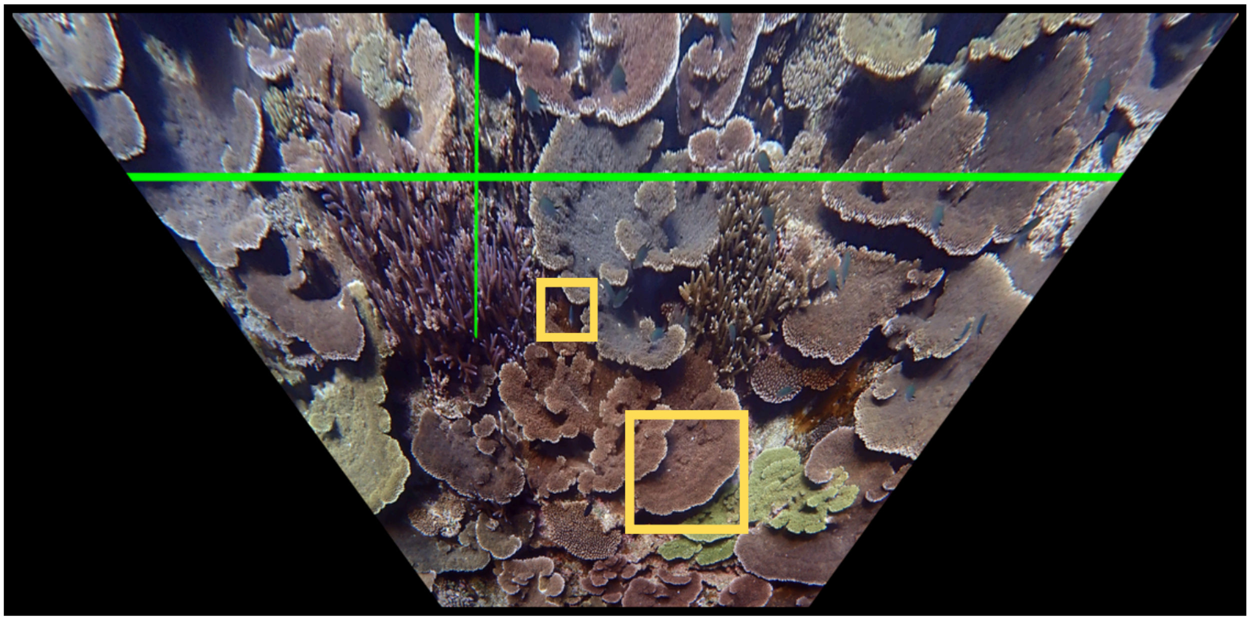

Supplementary Figure S11. The yellow squares represent the area captured by conventional small-size (0.25 m^2) (central) and large-size (1 m^2) (bottom right) photoquadrats in relation to the conservative expert-estimated 35 m^2 of reef captured by an ideal reefscape image. Note that the large-size photoquadrat highlights the annual growth bands of the *Acropora hyacinthus* colony that were used to derive the alternative 42 m^2 scale for this image.

Representative failure cases of the 19 images that were not fully processed by the *ReScape* algorithm, as shown in Supplementary Fig. S12: the first image in each row represents a reefscape image that aborted while being processed by the *ReScape* algorithm.

The first row in Supplementary Fig. S12 represents the case in (a) where excessive shadowing along the border of the image produced a large region of zero-edge density (not shown), which the Otsu thresholding classifies as the water column in the reef region (not shown), and therefore interferes with the Theil-Sen estimator when regressing the horizon (b), which was responsible for 26% of the processing failures.

The second row in Supplementary Fig. S12 represents the case where an excessive camera-roll angle in (c) caused the y-intercept of the horizon line to not update properly, which turned negative (cannot be shown), which was responsible for 26% of the processing failures.

The third row in Supplementary Fig. S12 represents the case in (d) where no candidate vanishing points were detected because of the inherent difficulty of texture analysis in complex imagery (i.e., none of the detected angle-filtered-grouped lines produced intersections that lay on the horizon line; not shown), which was responsible for 16% of processing failures.

The fourth row in Supplementary Fig. S12 represents the case in (e) where the image is out of focus which lowers the ability of the Canny-edge detector to detect the edges of objects in the scene. Out-of-focus images also cause substantial noise (i.e., many black pixels are present in the reef region, which attempts at morphological closing were not able to correct) in the Otsu-thresholded image (f), interfering with the Theil-Sen estimator during horizon-line regression (g), which was responsible for 11% of processing failures.

The fifth row in Supplementary Fig. S12 represents the case in (h) where lens flare from the sun causes excessive edges to be detected in the water-column region (not shown), which were then classified as reef during Otsu thresholding (i), interfering with the Theil-Sen estimator during horizon-line regression (j), which was responsible for 11% of the processing failures.

The sixth row in Supplementary Fig. S12 represents the extreme case in (k) where there is very high visibility in the background of the image, which causes the demarcation of the edge-pixel density to be less pronounced (not shown), resulting in no clear distinction for the horizon line in the Otsu-thresholded image (l), which produces an erroneous horizon line by the Theil-Sen estimator (m), which was responsible for 5% of processing failures.

The seventh row in Supplementary Fig. S12 represents the case in (n) where the sandy substrate produced very few texture features (o) and very few angle-filtered-grouped lines, none of which qualified as a candidate vanishing line (p). This processing error case was responsible for 5% of processing failures.

We recommend that users avoid these reefscape image compositions to further improve the processing rate of the *ReScape* algorithm.

ReScape's Representative Failure Cases

Explanation	Images (#)
Excessive shadowing along image borders interferes with the horizon-line regression	5
Excessive camera-roll angle causes the horizon-line y-intercept to not update properly	5
No candidate vanishing points detected despite expected processing behavior	3
Produced a poor Otsu-threshold image because the image is out of focus which interferes with the horizon-line regression	2
Sun glare on lens produced edges that interfered with the horizon-line regression	2
Severe case of improved visibility in the background of the image causing low horizon-line	1
No candidate vanishing points detected because the scene is primarily sand and did not produce many edges or lines	1

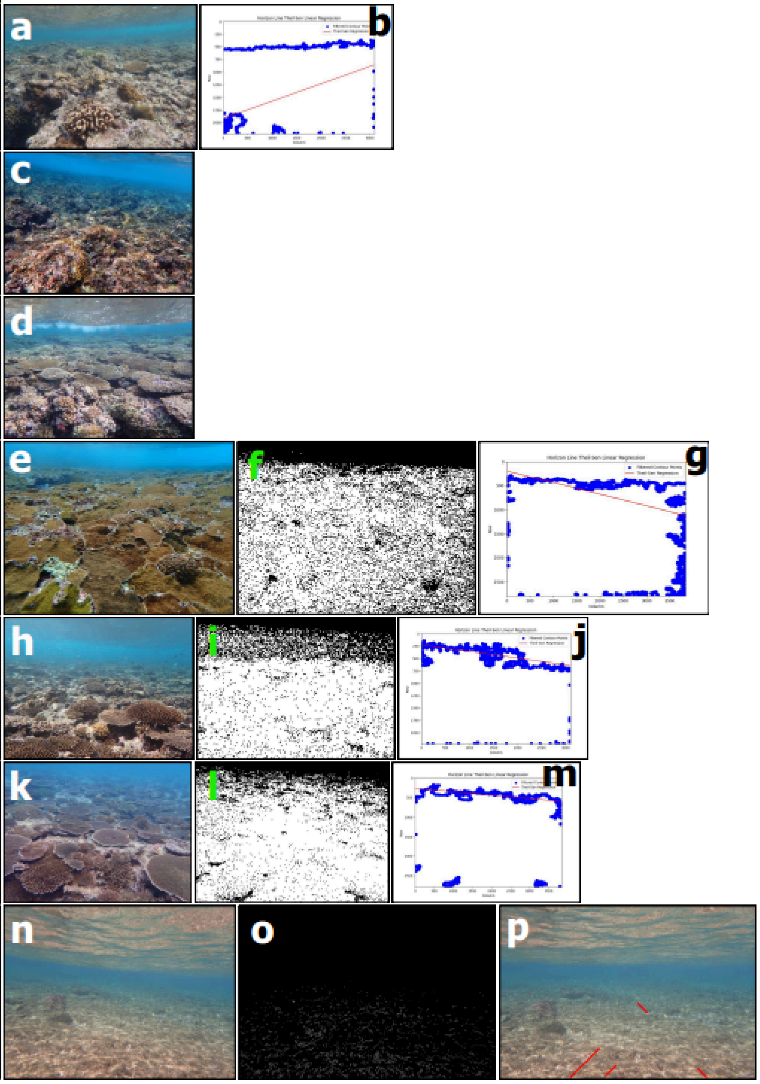

Supplementary Figure S12. Supporting visual for the *ReScape* algorithm's representative failure cases.
